# The structural basis of aldo-keto reductase 1C3 inhibition by 17α-picolyl and 17(*E*)-picolinylidene androstane derivatives

**DOI:** 10.1080/14756366.2025.2551979

**Published:** 2025-09-04

**Authors:** Jovana J. Plavša-Puž, Jiří Brynda, Jovana J. Ajduković, Sofija Bekić, Andjelka Ćelić, Pavlína Řezáčová, Jana Škerlová, Edward Petri

**Affiliations:** ^a^ Department of Biology and Ecology, University of Novi Sad, Novi Sad, Serbia; ^b^ Institute of Organic Chemistry and Biochemistry, The Czech Academy of Sciences, Prague, Czech Republic; ^c^ Department of Chemistry, Biochemistry and Environmental Protection, University of Novi Sad, Novi Sad, Serbia

**Keywords:** AKR1C3, prostate cancer, molecular docking, CYP17, protein X-ray crystallography

## Abstract

Human aldo-keto reductase 1C3 (AKR1C3) is a steroid modifying enzyme involved in cancer progression. Here, A-ring modified 17α-picolyl and 17(*E*)-picolinylidene androstane derivatives are shown to inhibit AKR1C3 activity *in vitro*. None of the androstane derivatives have off-target affinity for the androgen receptor, based on a fluorescence assay in yeast cells. The X-ray structure of AKR1C3 in complex with the strongest inhibitor, a 17α-picolyl androstane with a C3-oxime modification, was determined at 1.7 Å resolution. Based on this crystal structure and molecular docking, inhibition of AKR1C3 by the 17α-picolyl or 17(*E*)-picolinylidene derivatives depends on interactions between the C3 modification and the NADP^+^ cofactor, while the C17α-picolyl or C17-picolinylidene group anchors the inhibitor to AKR1C3. Because one AKR1C3 inhibitor identified here was also previously reported to inhibit CYP17, it may be possible for future researchers to design dual AKR1C3/CYP17 inhibitors based on a steroid scaffold for potential treatment of advanced prostate cancers.

## Introduction

Human aldo-keto reductase 1C3 (AKR1C3), also known as 17β-hydroxysteroid dehydrogenase 5 (17βHSD5) is an NAD(P)H dependent oxidoreductase involved in the conversion of weaker C17-keto androgens and oestrogens into their more potent C17-hydroxyl forms^1–4^. AKR1C3 is involved in regulation of androgen and oestrogen receptor activity and has been proposed as a target for the treatment of breast and prostate cancers^1^^,^^5–8^. In acute myeloid leukaemia (AML), AKR1C3 also functions as a prostaglandin D2 11-ketoreductase involved in AML progression. Because a steroidal AKR1C inhibitor, medroxyprogesterone acetate (MPA), stimulates the differentiation and apoptosis of AML leukaemia cell lines, AKR1C3 could also be a target for treatment of AML^9–11^. In addition to its role in cancer progression, AKR1C3 has been implicated in the development of resistance to chemotherapeutics for several cancer types^6^^,^^12–14^. Thus, studies have focused on identification of small-molecule inhibitors of AKR1C3 for the treatment of various cancers, especially for adjuvant therapy in combination with other anti-cancer drugs^6^^,^^15–20^.

Androgen receptor (AR) signalling plays a critical role in the development and progression of prostate cancer, and reduction of androgen levels is an important treatment strategy^21^^,^^22^. For example, inhibition of 17α-hydroxylase-17,20-lyase (P450 17A1 or CYP17A1), an enzyme that is essential for androgen synthesis, is the main mechanism of action of the prostate cancer drug abiraterone^23^^,^^24^. Abiraterone, a 17–(3-pyridinyl) androstane derivative, inhibits CYP17A1 with excellent efficacy, and is in clinical use for the treatment of advanced prostate cancers^25^^,^^26^. However, prostate cancer cells rapidly become resistant to abiraterone, due to re-activation of AR signalling via increased androgen levels and AR activation^22^^,^^27^. Previous studies show that abiraterone-treated prostate cancer cell lines overexpress AKR1C3^12,^^27^. Similarly, in abiraterone-resistant prostate cancer cells, AKR1C3 is strongly overexpressed and is associated with increased intracrine androgen levels. Moreover, overexpression of AKR1C3 induces resistance to abiraterone in prostate cancer cells, while AKR1C3 knockdown re-sensitizes these cells to abiraterone^13^^,^^15^. AKR1C3 activity is also associated with development of resistance in prostate cancer cells treated with the AR antagonist enzalutamide^28^^,^^29^. Treatment of abiraterone-resistant prostate cancer cells with indomethacin, a small-molecule AKR1C3 inhibitor, restores and even increases the sensitivity of prostate cancer cells to abiraterone therapy, while reducing intracrine androgen levels and AR transcriptional activity^15^^,^^30^. Because of this, several reports have proposed adjuvant inhibition of AKR1C3 in combination with approved treatments of prostate cancer and other cancers^29–31^. Because AKR1C3 metabolises chemotherapeutic drugs, leading to resistance; combining AKR1C3 inhibitors with chemotherapy drugs can enhance their cytotoxic effects, potentially improving treatment outcomes^14^^,^^16^^,^^32^. Thus, for adjuvant therapy, AKR1C3 inhibitors do not necessarily have to be cytotoxic to cancer cells on their own; but instead would ideally prevent metabolism and improve the cytotoxic and antiproliferative activity of other anti-cancer drugs.

We and others have shown that 17α-picolyl and 17(*E*)-picolinylidene androstane derivatives have significant anti-cancer activity against several human cancer cell lines, including prostate cancer cells^23^^,^^33^^,^^34^. 17(*E*)-picolinylidene androstane derivatives in particular have also shown potential as inhibitors of CYP17A1^23^^,^^24^. Our previous work suggests that steroidal derivatives with heterocyclic modifications could be inhibitors of AKR1C enzymes^19^. Because of their anticancer properties, we were particularly interested in investigating 17α-picolyl and 17(*E*)-picolinylidene androstane derivatives as AKR1C3 inhibitors. Compounds tested in the present study were chosen from our previous work based on their anti-proliferative properties against human cancer cell lines and/or ability to inhibit CYP17 or other steroidogenic enzymes (see Table 1). Several 17α-picolyl or 17(*E*)-picolinylidene derivatives chosen for the present study displayed cytotoxicity against PC3 prostate cancer cells (compounds **2**, **3, 4** and **6**), MCF7 breast cancer cells (compound **5** and **6**) or A549 lung cancer cells (compounds **7** and **8**); while two compounds (compounds **2** and **4**) were shown to be inhibitors of CYP17^23^^,^^33^^,^^34^ (see Table 2). In the present study, this series of previously synthesised and fully chemically characterised 17α-picolyl and 17(*E*)-picolinylidene androstane derivatives were tested for ability to inhibit AKR1C3 activity *in vitro*. The structural basis of the top AKR1C3 inhibitor was determined by protein X-ray crystallography to provide a framework for *in silico* studies and design of future compounds. Using this X-ray structure, the molecular basis of AKR1C3 inhibition by 17α-picolyl and 17(*E*)-picolinylidene androstane derivatives was then analysed by molecular docking simulations. Because compounds intended for the treatment of androgen-dependent cancers should not activate the androgen receptor, the 17α-picolyl and 17(*E*)-picolinylidene androstane derivatives were also tested for binding to the ligand binding domain of AR expressed in yeast cells.

**Table 1.** Structures of 17-picolinylidene and 17α-picolyl compounds used in the present study.

Structure		Compound name	Reference
1	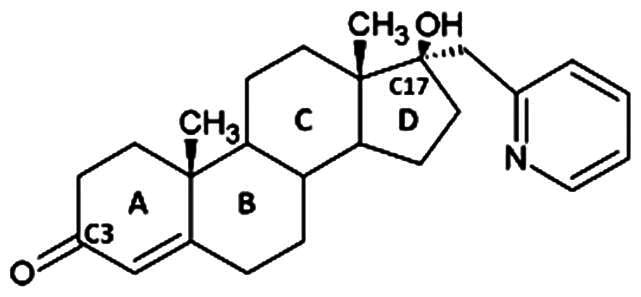	17β-hydroxy-17α-picolyl-androst-4-en-3-one	Compound 15 in Djurendjic et al. 2008^33^
2	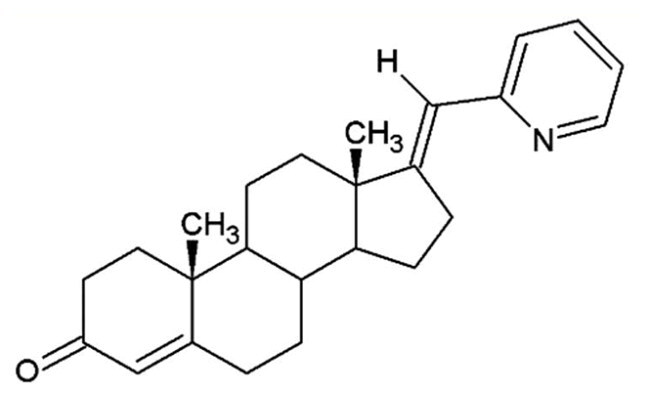	17-picolinylidene-androst-4-en-3-one	Compound 18 in Djurendjic et al. 2008^33^
3	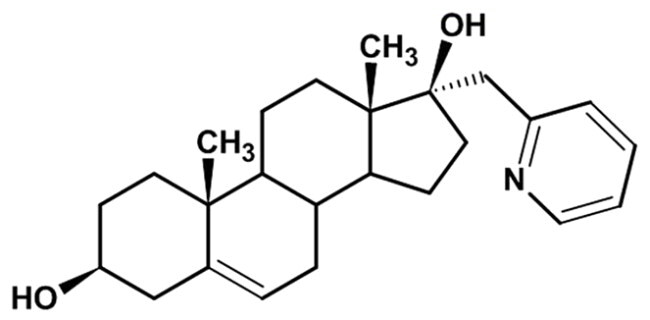	3β,17β-dihydroxy-17α-picolyl-androst-5-ene	Compound 1 in Djurendjic et al. 2008^33^
4	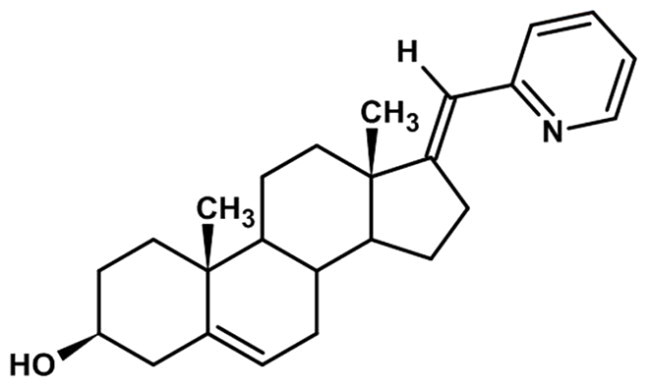	3β-hydroxy-17-picolinylidene-androst-5-ene	Compound 3 in Djurendjic et al. 2008^33^
5	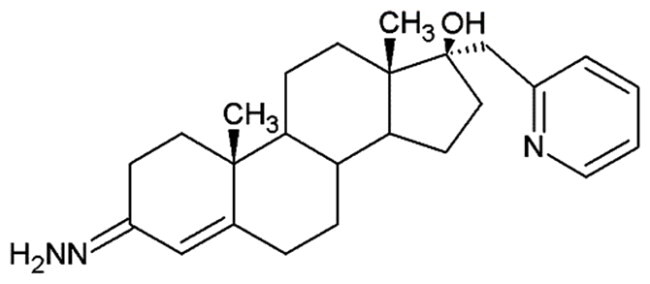	17β-hydroxy-17α-picolyl-androst-4-en-3-on hydrazone	Compound 3 in Ajdukovic et al. 2013^23^
6	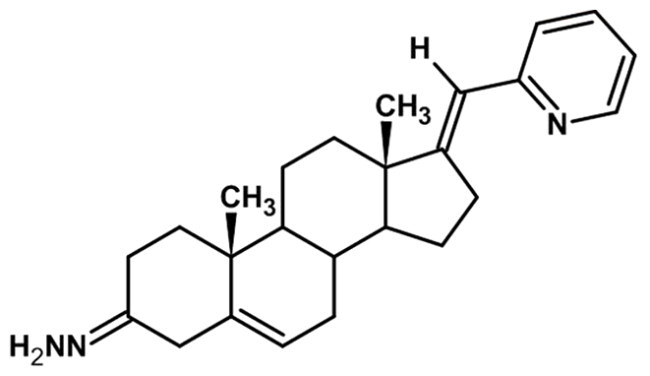	17(E)-Picolinylidene-androst-4-en-3-on hydrazone	Compound 4 in Ajdukovic et al. 2013^23^
7	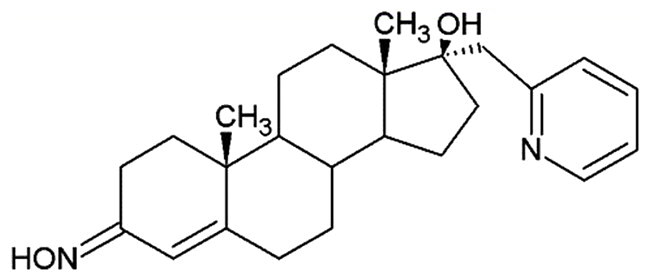	17β-Hydroxy-17α-picolyl-androst-4-en-(Z)-one oxime	Compound 3 in Ajdukovic et al. 2021^34^
8	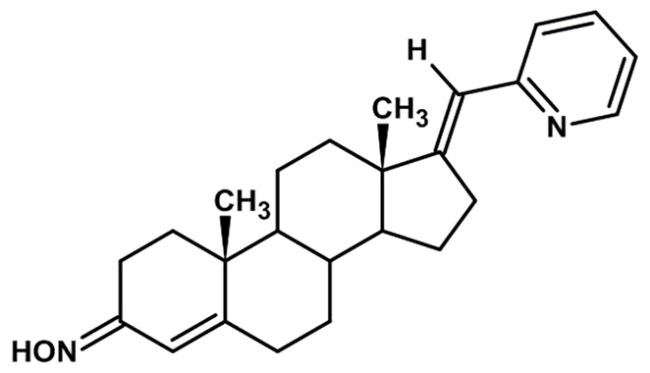	(17E)-Picolinylidene-androst-4-en-(E)-one oxime	Compound 12 in Ajdukovic et al. 2021^34^

The synthesis and chemical characterisation of compounds **1–8** are described in Supplementary material and in the listed references. For visualisation purposes, the locations of steroidal rings A, B, C and D, as well as carbon-17 (C17) and carbon-3 (C3) are labelled on compound **1**.

**Table 2.** AKR1C3 inhibition potential of test compounds (**1–8**).

		Cytotoxicity IC50 (µM)*								AKR1C3		
Compound	vLogP	PC3	MCF-7	MDA-MB-321	HT-29	A549	HeLa	MRC-5	CYP17A1*IC50 (µM)	%inhibition	IC50 (µM)	Docking energy (kcal/mol)
**1**	3.32	>100	>100	>100	–	–	–	>100		75.65 ± 1.89	31.69 ± 12.5	−13.7
**2**	3.83	12.9	>100	>100	–	–	–	>100	2.5 ± 1.4	60.12 ± 1.78	16.17 ± 1.52	−13.3
**3**	3.28	22.3	>100	3.8	>100	–	26.9	>100		49.43 ± 2.09	12.09 ± 4.16	−11.8
**4**	3.89	10.1	>100	>100	–	–	–	>100	8.2 ± 1.8	14.32 ± 2.10		−9.3
**5**	3.33	>100	6.3	32.5	>100	–	>100	>100		17.17 ± 1.85		−12.7
**6**	3.86	5.9	13.6	17.1	51.7	–	>100	>100		8.34 ± 1.73		−9.3
**7**	3.07	>100	>100	38.9	>100	52.4	>100	–		84.93 ± 5.62	14.44 ± 3.68	−14.3
**8**	3.68	>100	41	47.3	4.4	1.5	>100	>100		40.01 ± 4.75		−9.4
ibuprofen	Reference AKR1C3 inhibitor									75%	∼10 µM	
*References^23^^,^^24^^,^^33^^,^^34^												

Calculated values for percent inhibition (% inhibition) of AKR1C3 reduction of 9,10-phenanthrenequinone by test compounds **1–8** at final concentrations of 6.25 µM are shown along with IC50 values for compounds **1, 2, 3** and **7**. For comparison, AKR1C3 binding energies (kcal/mol) for compounds **1–8** predicted by molecular docking in Autodock Vina are shown in the same table. Published cytotoxicity data^23^^,^^33^^,^^34^ for **1–8** against human cell cancer lines and non-cancerous MRC-5 cells area shown, as well as IC50 values for compounds **2** and **4** for inhibition of CYP17^24^, and calculated LogP values. LogP values were calculated from the Molecular Lipophilicity Potential (MLP) using VEGA (https://www.ddl.unimi.it/vegaol/vlogp.htm)^69^.

## Materials and methods

### Chemical compounds

The synthesis and full chemical characterisation of compounds **1**–**8** has been published previously as described in Table 1, and is provided in Supplementary material.

### Expression and purification of recombinant human AKR1C3

Expression and purification of human AKR1C3 was performed as described^19^^,^^35^. Plasmid DNA encoding N-terminal 6 × His-tagged human AKR1C3 in a pET28b(+) expression vector was obtained from professor Chris Bunce (University of Birmingham, UK) for expression in BL21 (DE3) *E. coli* cells^9^. Chemically competent BL21 (DE3) *E. coli* cells were transformed with pET28-AKR1C3 and grown at 37 °C in LB media supplemented with 50 µg/mL kanamycin. AKR1C3 expression was induced at a cell density of OD_600_ = 0.5 − 0.6 with 1 mM IPTG (isopropyl β-D-1-thiogalactopyranoside), and cells were grown for 18 h at 20 °C before harvesting by centrifugation. Cell pellets were resuspended in buffer A (25 mM sodium phosphate, 500 mM NaCl, pH 8.0) supplemented with lysozyme (1 mg/mL) and stored at −80 °C. Cells were lysed by freeze-thaw cycles followed by sonication, and resulting lysate clarified by centrifugation at 13,000 × g for 45 min at 4 °C. Clarified supernatant was applied to a 1 ml HisTrap (GE HealthCare, USA) column packed with nickel sepharose and pre-equilibrated in 10 column volumes (CV) buffer A. The column was washed in 20 CV buffer A. AKR1C3 was then eluted in 5 CV buffer A with 500 mM imidazole and applied to a Bio-Rad P10 desalting column for buffer exchange into 25 mM sodium phosphate, 150 mM NaCl, 10% glycerol, pH 8.0. Protein purity was monitored by SDS-PAGE with Coomassie staining and protein concentration was quantified by a Bradford colorimetric assay. Purified AKR1C3 used for activity assays was stored at −80 °C in 25 mM sodium phosphate, 150 mM NaCl, pH 8.0, supplemented with 50% glycerol.

### Measurement of AKR1C3 enzymatic activity

AKR1C3 enzymatic activity was measured as described^19^. For reactions, test compounds, or ibuprofen (a positive control, known AKR1C3 inhibitor^20^) were added at a final concentration of 6.25 µM in 100 µL reactions containing 100 mM potassium phosphate buffer pH 6.0, 0.2 mM NADPH and 40 µg/mL AKR1C3 enzyme and incubated for 15 min at 37 °C. Test compounds were dissolved in DMSO such that the final DMSO concentration in each reaction was 2%. Reactions were conducted under initial velocity conditions, and were initiated by addition of 9,10-phenanthrenequinone (PQ), an AKR1C3 substrate, at a final concentration of 0.39 µM (corresponding to the Km of PQ for AKR1C3), and monitored by measuring the decrease in NADPH absorbance at 340 nm for 30 min in 96-well microplates (Carl Roth, Germany) using a Thermo Scientific Multiscan GO spectrophotometer^19^. Absorbance values were recorded every 30 s. The first 10 min of each reaction were analysed by linear regression (GraphPad Prism version 6.01, GraphPad Software, San Diego, California, USA, www.graphpad.com) to obtain corresponding slope values for absorbance (340 nm) vs. reaction time data. PQ-only control reactions were conducted in reaction buffer containing a final concentration of 2% DMSO. The slope obtained for PQ-only control reactions was defined to represent 100% AKR1C3 activity. Blank reactions were measured in the absence of enzyme for normalisation of the data. The percent inhibition (% inhibition) for each test compound was then calculated as:

$$\%\text{ inhibition }\left(\text{compound}\right)\;=\text{ 1}00\;-\;\left[\text{slope }\left(\text{PQ }+\text{ compound}\right)/\text{slope }\left(\text{ PQ‐only}\right)\;\times \text{ 1}00\right]$$

where slope (PQ + compound) corresponds to the slope of the reaction in the presence of PQ and test compound, and slope (PQ-only) corresponds to slope of the reaction with PQ and without inhibitor (test compound). Percent inhibition of AKR1C3 activity for test compounds was compared with that obtained for ibuprofen and PQ-only. For compounds **1** and **7**, dose-response curves were obtained by measuring the effect of increasing inhibitor concentrations from 0 to 500 µM (0.7, 2, 3, 7, 12, 25, 50, 100, 200, and 500 µM) on PQ reduction by AKR1C3. For analysis, values for ε_NADPH_ of 6.22 mM/cm and a path length of l = 0.562 cm were used. IC50 values were calculated by fitting the data to a four-parameter logistic sigmoidal dose-response curve using the program GraphPad. All results shown are mean values from three experiments. For compounds **2** and **3**, dose-response curves were obtained by monitoring changes in NADPH fluorescence over time during reduction of 9,10-phenanthrenequinone following treatment with 0, 0.78, 1.56, 3.12, 6.25, 12.5, 25, 50, 100 and 133 µM of compound **2** or **3** (see Figure S1). Reactions contained human AKR1C3 (80 µg/mL), 250 µM NADPH and 4 µM PQ in 100 mM potassium phosphate buffer pH 6.0. Enzyme and compounds were preincubated for 15 min at 37 °C and reaction was initiated by addition of NADPH and substrate. Fluorescence was measured in kinetic mode every 30 s for 10 min at 37 °C using excitation/emission wavelengths of 340/460 nm in a Fluoroskan Ascent FL fluorimeter. Enzyme activity was expressed relative to uninhibited control (defined as 100% activity) based on the slope of fluorescence vs. time data. IC50 values were determined using an online tool https://ic50.org/, yielding calculated IC50 values of 16.17 ± 1.52 µM for compound **2** and 12.09 ± 4.16 µM for compound **3**.

### Crystallisation of AKR1C3 in complex with inhibitors using in situ proteolysis of the N-terminal His tag

Protein expression and purification for crystallisation was done as described for the enzymatic assays, except that the final buffer was 10 mM potassium phosphate pH 7.0, 1 mM EDTA, 1 mM DTT^36^. Purified AKR1C3 supplemented with 1.2 mM NADP^+^ (Carl Roth, Germany) was concentrated up to 38 mg/mL. Compound **1**, **2** or **7** was added to the protein solution at a final concentration of 5 mM from 100 mM stock in 100% DMSO, incubated on ice for 30 min and cleared by centrifugation. Screening was performed at 18 °C by sitting-drop vapour diffusion using Gryphon (Art Robbins Instruments, USA) and Oryx8 (Douglas Instruments, UK) crystallisation workstations in 96-well sitting drop MRC 3-well plates (Jena Bioscience, Germany). The commercial protein crystallisation screen Morpheus (Molecular Dimensions, UK) was used for crystallisation trials. Immediately prior to crystallisation trials, bovine thrombin (Sigma Aldrich, USA) was added for *in situ* proteolysis of the N-terminal His tag at a AKR1C3:thrombin molar ratio of 280:1. As previously reported, addition of thrombin at this stage of crystallisation resulted in improved AKR1C3 crystal morphology and diffraction properties^36^. A total volume of 300 nL of a mixture of protein with inhibitor compound and thrombin, and precipitant solutions in 2:1 and 1:2 volume ratios was equilibrated against a 30 µL reservoir solution. The crystallisation process was monitored using a Minstrel DT UV automated imaging system with a Gallery DT plate hotel (Rigaku, Japan) and an Olympus SZX10 optical microscope (Olympus, Japan). In the presence of compound **1** or compound **2**, crystals of AKR1C3 were obtained in a precipitant solution containing 10% w/v PEG 20 000, 20% v/v PEG MME 550, 0.02 M of each alcohol (1,6-hexanediol, 1-butanol, (RS)-1,2-propanediol, 2-propanol, 1,4-butanediol, 1,3-propanediol) and 0.1 M MES/imidazole pH 6.5 as a buffer. The best AKR1C3 crystals in the presence of compound **7** were obtained in a precipitant solution containing: 10% w/v PEG 20 000, 20% v/v PEG MME 550 and 0.03 M each of sodium fluoride, sodium bromide and sodium iodide, using 0.1 M MES/imidazole pH 6.5 as a buffer.

### X-ray diffraction data collection and processing

Crystals of AKR1C3 grown in the presence of compounds **1**, **2** or **7** were harvested and flash-frozen in liquid nitrogen without additional cryoprotection^37^. Diffraction quality was tested at 100 K on an in-house MicroMax-007 HF Microfocus X-ray generator with a VariMax VHF Arc) Sec confocal optical system (Rigaku, Japan) equipped with an AFC11 partial-four-axis goniometer (Rigaku, Japan), a PILATUS 300K detector (Dectris, Switzerland) and a Cryostream 800 cryocooling system (Oxford Cryosystems, UK). Diffraction data was collected at 100 K in-house (for crystals of AKR1C3 grown in the presence of compound **7**), or at MX 14.1 operated by the Joint Berlin MX-Laboratory at the BESSY II electron-storage ring in Berlin-Adlershof, Germany^38^^,^^39^. Diffraction data were processed using XDS^40^.

### Structure solution

The structures of human AKR1C3 crystallised in the presence of compounds **1**, **2** and **7** were solved by molecular replacement with the program PHASER in CCP4, using the structure of human AKR1C3 in complex with a steroidal inhibitor as the search model (PDB: 1ZQ5)^41–43^. While crystals of AKR1C3 could be grown in the presence of compounds **1** and **2**, no electron density for a bound ligand was visible upon refinement. In contrast, clear electron density for compound **7** was immediately visible following molecular replacement and initial refinement. Model refinement was performed using REFMAC5^41^ in the CCP4 suite^40^ coupled with manual model building and refinement in the program COOT^43–45^. Translation, rotation, and screw-rotation (TLS) refinement was used throughout refinement, with 8 TLS groups defined as follows: for chain A residues 6–124, 125–137, 138–306, 307–323; for chain B residues 6–124, 125–153, 154–305, and 306–323^46^. Coordinates for compound **7** were added during the final stages of refinement using the tools available in COOT. The structure of AKR1C3 in complex with NADP^+^ and compound **7** has been deposited in the Protein Data Bank (PDB) (PDB ID 8RRJ).

### Molecular docking

#### Preparation of AKR1C3 receptor coordinates

Coordinates from the structure of AKR1C3 in complex with NADP^+^ and compound **7** (PDB: 8RRJ) were used as “receptor” for molecular docking simulations. Ligands were removed using a text editor, while the NADP^+^ cofactor and bound waters were retained. Hydrogen atoms were added to the receptor and Gasteiger partial charges were assigned using the script “receptor.c” in the program VEGAZZ 3.1.0^47^^,^^48^. Non-polar hydrogen atoms were merged and receptor coordinate files were converted to PDBQT format for molecular docking simulations in Autodock Vina^49^.

##### Preparation of ligand coordinates

Based on the 3D structure of compound **7**, structural models for test compounds **1–8** were created in the program Avogadro 1.1.2, an open-source molecular builder and visualisation tool (http://avogadro.cc/)^50^. Hydrogen atoms were added and geometry optimised using an MMF94s force field with 500 steps of conjugate gradient energy minimisation and 500 steps of steepest descent energy minimisation with a convergence setting of 10 × 10^−7^. Gasteiger partial charges were assigned and nonpolar hydrogen atoms were merged using the script “ligand.c” in VEGAZZ 3.1.0 to create PDBQT files for molecular docking simulations in Autodock Vina^49^^,^^51^.

##### Molecular docking in Autodock Vina

Molecular docking screening was conducted in Autodock Vina using the PyRx screening tool and the following parameters: exhaustiveness = 8, centred at x = −12, y = 31 and z = −7 using a search space of 25 × 25 × 25 Å^51^. Results were visualised using the open-source program PyMOL v0.99 and compared with the structure of AKR1C3 in complex with NADP^+^ and compound **7**^52^.

#### Cross-docking simulations in Autodock Vina

Cross-docking simulations were conducted using superimposed structures of AKR1C3 in complex with non-steroidal anti-inflammatory drug (NSAID) inhibitors ibuprofen (PDB 3R8G) and flufenamic acid (PDB 1S2C) or compound **7** (PDB 8RRJ) as “receptors” in Autodock Vina. Ibuprofen, flufenamic acid and compound **7** were “redocked” into their respective native crystal structures or cross-docked against the other two AKR1C3 conformations. Receptors and ligands were prepared as described above. Docking was conducted with an exhaustiveness setting of 16 and a search space of 25x25x25 Å centred at the following coordinates: for AKR1C3-ibuprofen 3R8G center_x = −12.605581148, center_y = 31.3123440954, center_z = −6.82004214437; for AKR1C3-flufenamic acid 1S2C center_x = −11.937326153, center_y = 31.4814683288, center_z = −8.09784988963; for AKR1C3-7 center_x = −12.5864986708, center_y = 32.2920257137, center_z = −6.40682483789. Root mean square deviation of atomic positions (RMSD) between predicted docked ligand poses (output from Autodock Vina) and native ligand poses (coordinates taken from 3R8G, 1S2C or 8RRJ) were calculated in Autodock Tools (MGLTools v 1.5.7). Results from docking simulations were analysed in PyMol and predicted binding energies (kcal/mol) were obtained for top ranking poses from Autodock Vina.

### Fluorescence assay for androgen receptor binding in yeast cells

The yeast strain, FY250 (MATα, *ura3-52*, *his32Δ00*, *leu2Δ1*, *trp1Δ6*) and plasmid construct pRF4-6-AR LBD-EYFP encoding the ligand binding domain (LBD) of the androgen receptor fused to enhanced yellow fluorescent protein (EYFP) used in the fluorescent cell assay were provided by dr. Blake Peterson, University of Kansas^53^. Yeast transformations were performed using a lithium acetate/polyethylene glycol procedure^54^. Yeast cells transformed with plasmid DNA were selected on tryptophan dropout agar plates after 3 days growth at 30 °C. Master plates were created from individual clones and stored at 4 °C. The fluorescence assay in yeast was optimised for testing steroidal derivatives binding to the oestrogen or androgen receptor. Selective media supplemented with 2% raffinose was inoculated with yeast cells and incubation was performed in a Biosan orbital shaker-incubator ES-20/60 until saturation. Cell growth was monitored by measuring optical density spectroscopically at 600 nm (OD_600nm_) using a Nicolet Evolution 100 UV-Vis spectrophotometer. Saturated pre-cultures were diluted at OD_600nm_ ∼ 0.1 in fresh medium of the same composition and incubation was continued with agitation and aeration at 30 °C for 2–3 generations. Expression of the AR LBD-YFP protein was induced in mid-log phase (OD_600nm_ ∼ 0.4–0.6) by addition of galactose (2% final concentration). Test compounds **1–8**, oestrone (a negative control AR ligand), androstenedione (a positive control AR ligand) or testosterone (a positive control AR ligand) were then added at a final concentration of 10 μM. Treated cells were incubated at room temperature for 15 h in the dark. Fluorescence measurements were conducted in triplicates in a 96-well format (Carl Roth, Germany) using 150 μL of intact cell suspensions. Growth medium served as a blank. Intensity of fluorescence was read on Fluoroskan Ascent FL fluorimeter with excitation and emission wavelengths set to 485 and 538 nm, respectively. To obtain a normalised fluorescence signal, fluorescence intensity was divided by cell optical density at 600 nm. Ligand binding affinity was expressed as fold fluorescence against the negative control. Histograms were generated using Origin Pro 8 software. Error bars represent propagated standard errors of the mean. For visualisation of individual cell fluorescence distribution and qualitative analysis 3 μL of concentrated cell suspension was observed under a fluorescence microscope Olympus BX51 using a FITC filter.

## Results and discussion

### Inhibition of AKR1C3 activity in vitro by compounds 1–8

**Figure 1.**
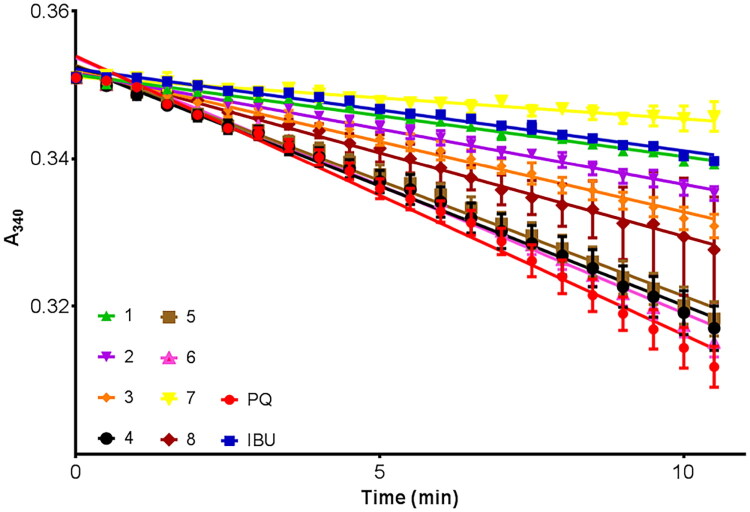
AKR1C3 reduction of 9,10-phenanthrenequinone (PQ) in the absence or presence of either test compounds (**1–8**) or ibuprofen (IBU) at a final concentration of 6.25 µM. The effect of test compounds or ibuprofen on AKR1C3 activity was measured by monitoring the decrease in NADPH absorbance at 340 nm over time. Data shown represent the mean of three experiments, fit by linear regression.

In the present study, a series of previously synthesised and characterised 17α-picolyl and 17(E)-picolinylidene androstane derivatives were tested for their ability to inhibit AKR1C3 activity *in vitro*. The ability of test compounds **1–8** to inhibit reduction of 9,10-phenanthrenequinone (PQ) by AKR1C3 was measured using a standard spectroscopic assay^19^^,^^55^^,^^56^. The assay monitors the decrease in absorption of NADPH at 340 nm, corresponding to consumption of NADPH cofactor during reduction of PQ by AKR1C3. Control reactions conducted in the absence of enzyme or without inhibitor were used to define 100% and 0% inhibition values, respectively. A known AKR1C3 inhibitor, ibuprofen (IBU), was included as a positive control, and resulted in ∼75% inhibition of AKR1C3 activity^56^. Test compounds or ibuprofen were added at a final concentration of 6.25 µM, and reactions were conducted under initial velocity conditions, by addition of PQ at a final concentration of 0.39 µM, which corresponds to the Km of PQ for AKR1C3. As can be seen in Figure 1, addition of compound **7** inhibited AKR1C3 reduction of PQ by **∼**85% compared to control reactions. Under the assay conditions used, compound **7** was a stronger inhibitor than ibuprofen, while compound **1** (75% inhibition) was as effective as ibuprofen. As summarised in Table 2, compound **2** (∼60% inhibition) was also identified as a potential AKR1C3 inhibitor, followed by compound **3** (∼49%) and compound **8** (∼40%).

While additional compounds would have to be tested to enable more reliable analysis of structure activity relationships, results from the present study do suggest that A-ring modified 17α-picolyl and 17(*E*)-picolinylidene androstane derivatives are promising candidates for the design of new AKR1C3 inhibitors. Moreover, 17α-picolyl derivatives were in all cases stronger AKR1C3 inhibitors than their otherwise identical 17-picolinylidene counterparts. This is supported by the following direct comparison of pairs of compounds where the only difference is a 17α-picolyl or 17-picolinylidene modification: 17α-picolyl compound **1** (∼75%) is more effective than 17-picolinylidene compound **2** (60%); 17α-picolyl **3** (∼49%) is better than 17-picolinylidene **4** (14%); 17α-picolyl **5** (∼17%) is better than 17-picolinylidene **6** (8%) and 17α-picolyl **7** (∼85%) is better than 17-picolinylidene **8** (40%). Analysis based on the C3 modification could also suggest several potential trends, especially if modifications are compared separately within picolyl and picolynilidene derivatives. Namely the 3-oxo in compound **1** is associated with stronger AKR1C3 inhibition than the otherwise identical compound **3** with a 3-hydroxyl modification (75% vs. 49%); while the 3-oxime modification in **7** and **8** is associated with much stronger AKR1C3 inhibition than analogous compounds **5** and **6** with a C3 hydrazone modification. Based on these results, it is tempting to speculate that AKR1C3 inhibition potential is more sensitive to the type of modification present on C3 of the steroid than the presence of either 17α-picolyl or 17-picolinylidene at C17.

In addition to the AKR1C3 inhibitory activity observed here, a 17(*E*)-picolinylidene derivative, compound **2**, was previously shown to inhibit CYP17 with an IC50 of 5.9 µM against its 17α-hydroxylase activity and 2.5 µM against its 17,20-lyase activity^23^^,^^24^ (Table 2). Compound **2** was also reported to be moderately cytotoxic to PC3 prostate cancer cells with an IC50 of 12.9 µM^23,^^33^ (see Table 2). Taken together, our *in vitro* assay results suggest that compounds **7**, **1** and **2** could all be used for the design of AKR1C3 inhibitors. In addition, although beyond the scope of the present study, results for compound **2** suggest that steroidal compounds may be used in the future for the design of dual AKR1C3/CYP17 inhibitors for use in the treatment of advanced prostate cancer^15^^,^^57^.

**Figure 2.**
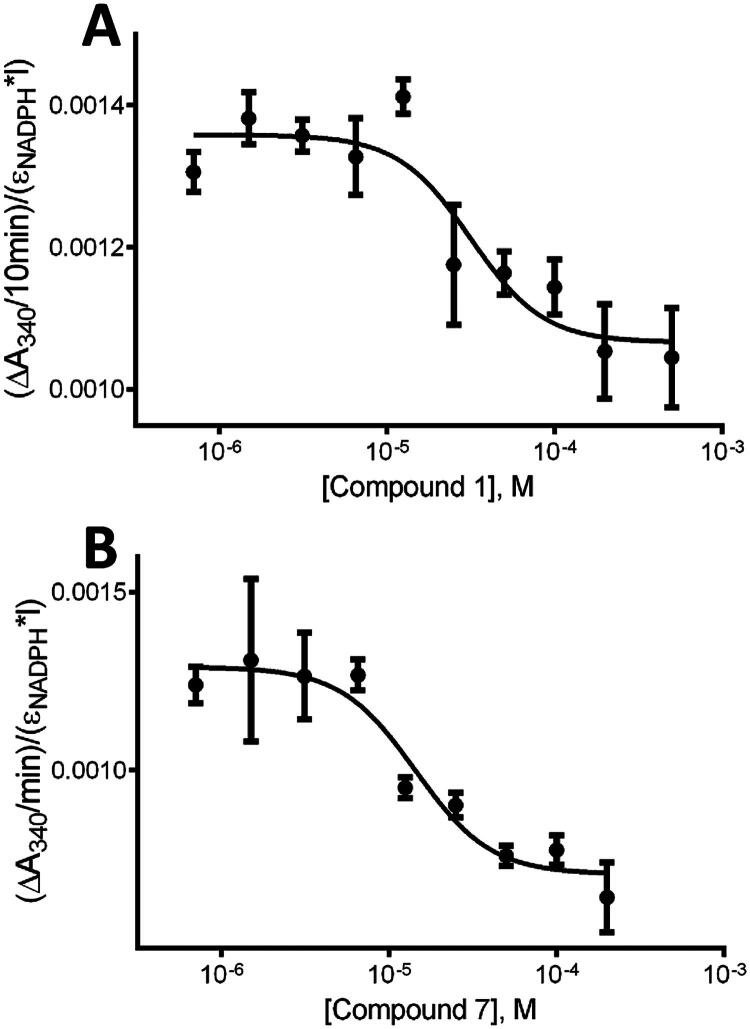
Effect of increasing concentrations of compounds **1** and **7** on AKR1C3 activity, represented by the decrease in NADPH absorption at A_340nm_ over time. Dose-response curves were obtained by measuring the effect of increasing concentrations of compound **1** (panel A) and **7** (panel B), from 0 to 500 µM (0.7, 2, 3, 7, 12, 25, 50, 100, 200, and 500 µM in 2% DMSO) on PQ reduction by AKR1C3. The concentration of PQ was constant at 0.39 µM, near the Km value obtained for AKR1C3 under the assay conditions used^19^. IC50 values were calculated by fitting the data to a four-parameter logistic sigmoidal dose-response curve (GraphPad). All results shown are mean values from three experiments.

To further validate their AKR1C3 inhibitory potential, dose-response curves were measured for compound **1** and compound **7**, the top two inhibitors identified in the screen. Based on the analysis of the dose response data, a calculated IC50 value of ∼32 µM was obtained for compound **1**, and ∼14 µM for compound **7** (Figure 2 and Table 2). Similarly, IC50 values were calculated from dose-response curves for compound **2** (16.17 ± 1.52 µM) and compound **3** (12.09 ± 4.16 µM) (see Figure S1 and Table 2). For comparison, an IC50 value of ∼10 µM has been reported for our positive control AKR1C3 inhibitor ibuprofen^56^.

### Affinity of the test compounds for the androgen receptor

**Figure 3.**
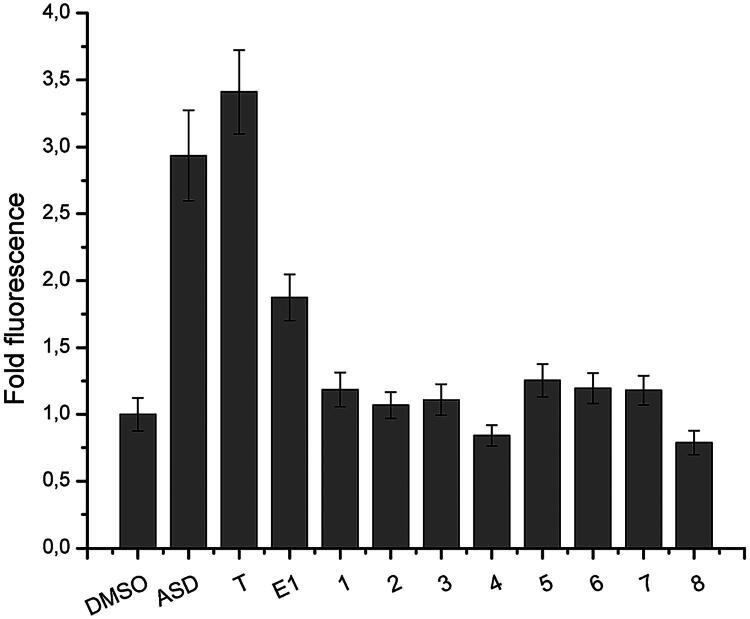
Fold fluorescence change in yeast cells expressing AR-LBD-EYFP upon treatment with test compounds. The fold fluorescence change in yeast cells expressing recombinant receptor binding domain of androgen receptor fused in frame with enhanced yellow fluorescent protein was measured by fluorescence spectroscopy, following treatment with test compounds (**1–8**) or control steroidal AR ligands. Androstenedione (ASD) and testosterone (T) were used as positive control AR ligands, while oestrone (E1) was used as a known low affinity AR ligand (negative control).

The affinity of compounds **1–8** for the androgen receptor (AR) was measured using a yeast-based fluorescence assay optimised for screening steroidal derivatives with oestrogen, androgen or glucocorticoid receptor binding properties^53^^,^^58^. For AR expression in yeast, a galactose-inducible plasmid (pRF4-6-AR-LBD-EYFP) encoding the ligand binding domain (LBD) of the androgen receptor (AR) fused to enhanced yellow fluorescent protein (EYFP) was used^58^^,^^59^. Expression of AR-LBD-EYFP in yeast cells was detected by fluorescence microscopy, and dose-dependent binding of a positive control AR ligand, testosterone, was measured. Test compounds **1–8** were then tested for relative affinity to the AR-LBD by evaluation of the fold fluorescence change between compound-treated, oestrone-treated (negative control), testosterone-treated (AR positive control ligand) and androstenedione-treated (ASD, AR positive control ligand) yeast cells expressing AR-LBD-EYFP, as previously described^58^. As seen in Figure 3, no compounds displayed affinity for AR, while testosterone and ASD treatment resulted in strong fluorescence signal corresponding to a 3 − 3.5 fold fluorescence increase.

### Crystal structure of AKR1C3 in complex with the NADP^+^ cofactor and compound 7

Based on *in vitro* AKR1C3 activity assays, compound **7** with both a C3-oxime and C17α-picolyl modification displayed the strongest overall AKR1C3 inhibition. To study the structural basis of AKR1C3 inhibition by 17α-picolyl androstane derivatives, we focused next on X-ray structure determination of the AKR1C3-NADP^+^ in complex with compound **7** (hereafter AKR1C3-**7**). The structure was determined at a resolution of 1.7 Å (data collection and refinement statistic are listed in Table 3) and is available in the PDB under accession code 8RRJ.

**Table 3.** Data collection and refinement statistics for the crystal structure of AKR1C3-NADP^+^ in complex with compound **7.**

Data collection statistics	
Space group	P1
Cell parameters a, b, c [Å]; a, β, γ [°]	41.17, 53.52, 75.62; 77.71, 85.61, 76.06
Wavelength [Å]	1.54187
Resolution [Å]	46.49–1.70 (1.74–1.70)
Unique reflections	55,988 (4,900)
Multiplicity	3.34 (1.86)
Completeness [%]	83.1 (98.1)
R_meas_ [%]^a^	9.9 (110.3)
CC_(1/2)_ [%]^b^	99.6 (47.5)
Average I/σ(I)	8.70 (1.01)
Wilson B [Å^2^]^c^	28.45
**Refinement statistics**	
Resolution range [Å]	46.49–1.70 (1.74–1.70)
No. of reflections in working set	54,868 (4,798)
No. of reflections in test set	1,120 (98)
R value [%]^d^	18.69 (30.5)
R-free value [%]^e^	22.12 (31.3)
RMSD deviation from ideal bond length [Å]	0.010
RMSD deviation from ideal bond angle [°]	1.733
Number of protein atoms	5,159
Number of water molecules	581
Number of other non-protein atoms	165
Mean B value [Å^2^]	23.6
Residues in Ramachandran favoured regions [%]^f^	97.2
Residues in Ramachandran allowed regions [%]^f^	100

Values in parentheses refer to the highest resolution shell. ^a^R_meas_ defined in Ref.^57^^,^^70^. ^b^CC_(1/2)_ is Pearson’s correlation coefficient determined on the data set randomly split in half^71^. ^c^Wilson B by the Sfcheck program from the CCP4 suite^72^. ^d^R-value = ǁ*F*_o_| – |*F*_c_ǁ/|*F*_o_|, where *F*_o_ and *F*_c_ are the observed and calculated structure factors, respectively. ^e^R_free_ is equivalent to the R-value but is calculated for 5% of the reflections chosen at random and omitted from the refinement process^73^. ^f^As determined by MolProbity^74^.

**Figure 4.**
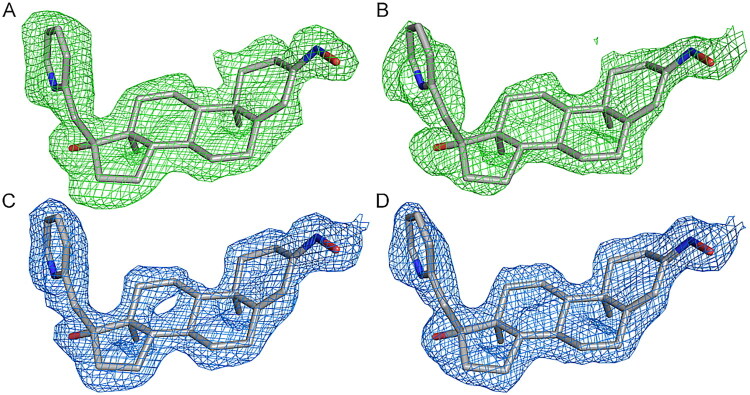
Electron density maps for compound **7**. The Fo-Fc difference electron density maps for compound **7** in active site A (panel A) and active site B (panel B) calculated before ligand modelling are shown as a green mesh, contoured at 2σ. Panels C and D show the final refined 2Fo-Fc electron density maps as a blue mesh contoured at 1σ for compound **7** in active sites A and B, respectively. The final refined coordinates of the ligands are shown in all panels for comparison. For the map of calculated molecular electrostatic potential for compound **7**, see Figure S2.

The asymmetric unit comprised two molecules of AKR1C3 with clear electron density for amino acids 6 − 323 and the NADP^+^ cofactor. The quality of the electron density suggested a higher flexibility of the regions between residues 320 and 323, and 125 and 137. Clear electron density for compound **7** was visible in the active sites of both AKR1C3 monomers (see Figure 4). The two AKR1C3 molecules have a very similar structure and superpose with an RMSD value of 0.191 Å for 312 pairs of Cα atoms. The location, binding geometry and conformation of compound **7** was identical in both monomers of AKR1C3.

**Figure 5.**
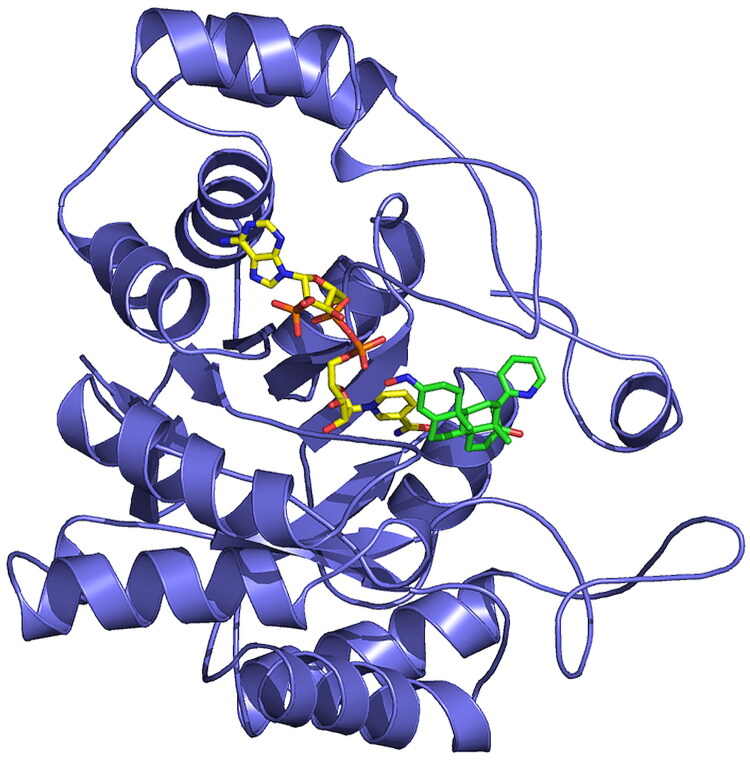
X-ray structure of AKR1C3 in complex with NADP^+^ and compound **7**. Chain B of the structure of AKR1C3 is shown in complex with cofactor NADP^+^ (yellow sticks) and compound **7** (green sticks).

Globally, the X-ray structure of AKR1C3-**7** (Figure 5) is very similar to other reported structures of AKR1C3, with an (α/β)8 triose-phosphate isomerase barrel motif present in all members of the aldo-keto reductase superfamily^4^^,^^20^. Consistent with this, the structure of AKR1C3 in complex with compound **7** can be aligned with the structure of AKR1C3 in complex with ibuprofen (PDB: 3R8G) with an RMSD of 0.254 Å^20^.

**Figure 6.**
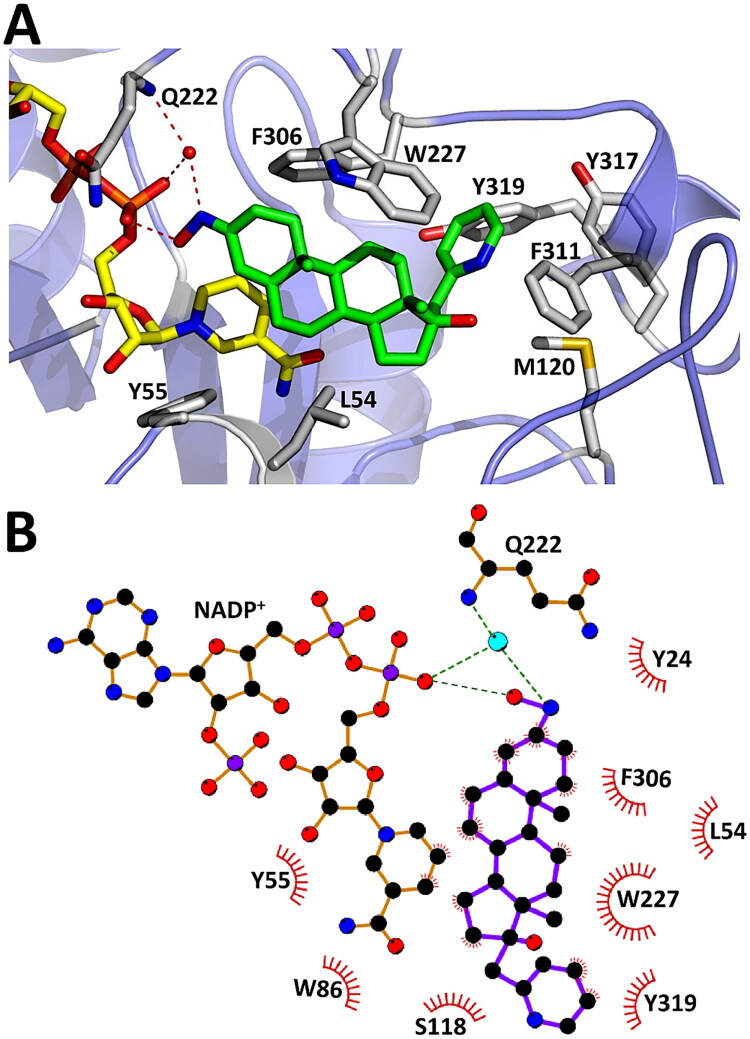
Molecular interactions between compound **7** and AKR1C3 ligand binding site. Panel A: Compound **7** (green) interacts with AKR1C3 residues (white sticks) corresponding to the steroid channel (W227, L54), SP1 (F306, F311, Y319) and SP2 (W227). The 3-oxime group of compound **7** forms a direct hydrogen bond between the oxime oxygen and O2N from NADP+ and a water-mediated hydrogen bond with NADP^+^. This same water also mediates a hydrogen bond between the oxime nitrogen with the main chain nitrogen of Q222. The C17 picolyl group interacts with SP1 (F311, Y319) and SP2 (W227) residues as well as with M120. Panel B: 2D representation of molecular interactions between AKR1C3 and compound **7**. Residues involved in compound **7** binding were identified using an automated program, LigPlot. Residues within 4 Å of compound **7** are shown.

The NADP^+^ cofactor is bound in the same location and conformation as in all other AKR1C3 structures determined to date. In the literature, the AKR1C3 ligand binding cavity has been defined to be composed of five key regions: an oxyanion site (Tyr55, His117 and NADP^+^), a steroid channel (Trp227 and Leu54), and three subpockets that are involved in ligand binding: SP1 (Ser118, Asn167, Phe306, Phe311 and Tyr319), SP2 (Trp86, Leu122, Ser129 and Phe311), and SP3 (Tyr24, Glu192, Ser221 and Tyr305)^1^^,^^20^. Compound **7** forms important interactions with regions of AKR1C3 shown to be essential for ligand recognition and binding, including: the oxyanion site, the steroid channel, and all three subpockets SP1, SP2, SP3 (Figure 6, panel A). In the Oxyanion site, the A-, B- and D-rings of compound **7** interact with the nicotinamide moiety of NADP^+^ and catalytic residues Y55 and H117, while the 3-oxime group (HON = C3) forms a hydrogen bond with an oxygen atom of the NADP^+^ phosphate group. In addition, the 3-oxime also participates in a water mediated hydrogen bond interaction with NADP^+^ and the main chain nitrogen of Q222, via an ordered water that is conserved in other AKR1C3 structures (e.g. 3R8G)^20^. In the steroid channel, numerous hydrophobic interactions were observed between compound **7** and a highly conserved tryptophan (W227), which lies parallel to the steroid core, supported by hydrophobic interactions with L54^1^. The C17 picolyl group of compound **7** projects deep into the SP1 subpocket, with specific interactions between the picolyl group, tyrosine Y319 and phenylalanine F311, as well as a less conserved methionine (M120) involved in binding the natural AKR1C3 inhibitor baccharin^60^. To provide a map of potentially important interactions between compound **7** and AKR1C3, a 2D protein-ligand interaction plot was also generated using the automated program LigPlot^61^^,^^62^. The plot highlights hydrophobic contacts between the steroidal core of compound **7** and the AKR1C3 steroid binding channel, while also identifying the locations of AKR1C3 interactions with the C17-picolyl group and the C3-oxime group (Figure 6, panel B).

**Figure 7.**
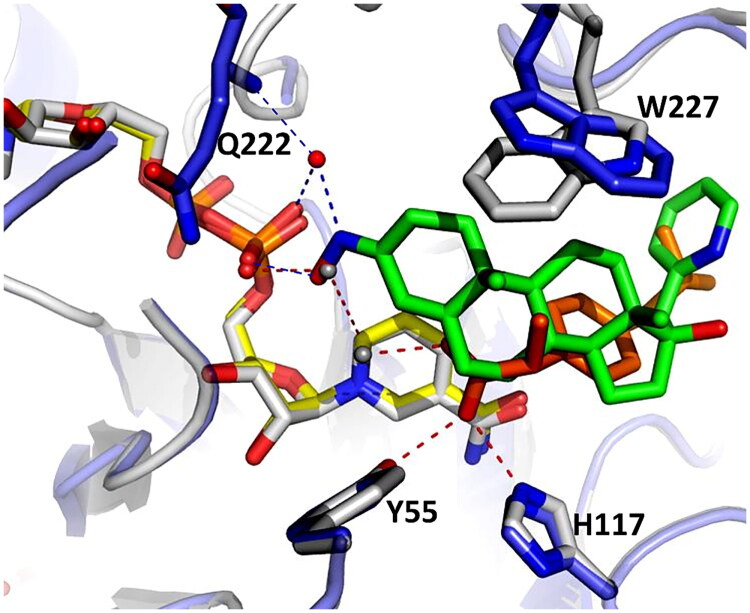
Comparison of structures of AKR1C3 in complex with compound **7** and ibuprofen. The structure of AKR1C3-**7** (blue) was aligned with the structure of AKR1C3-ibuprofen (white, PDB: 3R8G) with an RMSD of 0.254 Å. Compound **7** is shown in green and ibuprofen in orange. Select ordered waters (red for AKR1C3-**7**, white for AKR1C3-ibuprofen), the NADP^+^ cofactor (yellow for AKR1C3-**7**, white for AKR1C3-ibuprofen) and the relative positions of select residues important in ligand binding (W227) and catalysis (Y55, H117) are shown. Hydrogen bonds are shown in blue for AKR1C3-7 and red for AKR1C3-ibuprofen.

To better understand the structural basis of AKR1C3 inhibition by compound **7**, the structure of AKR1C3-**7** was also compared with the structure of AKR1C3-ibuprofen (PDB: 3R8G) (Figure 7)^20^. Overall, the binding position of ibuprofen is occupied by the B-, C- and D-rings of compound **7** in our structure, as well as a portion of the 17-picolyl group. The conserved tryptophan W227 appears to rotate in order to adjust to the presence of compound **7** or ibuprofen. In addition, the C3-oxime in our structure binds in the same position as an ordered water in the AKR1C3-ibuprofen structure, where it participates in direct and water-mediated hydrogen bonding with NADP^+^ (see Figures 6 and 7). However, the mechanism of inhibition may be somewhat different, since the bioactivity of ibuprofen appears to depend on interaction between its carboxylate and oxyanion site residues, Y55 and H117^20^, while compound **7** interacts more closely with the NADP^+^ cofactor.

In summary, based on analysis of the X-ray structure of AKR1C3 in complex with compound **7**, we propose that hydrogen bonding interactions between NADP^+^ and the C3-oxime are likely to be critical for AKR1C3 inhibition, while the C17-picolyl could serve to provide increased affinity due to hydrophobic interactions within the SP1 subpocket of the AKR1C3 ligand binding site.

### Cross-docking of ibuprofen, flufenamic acid and compound 7 into structures of AKR1C3

In addition to structural and experimental methods, we are also interested in using molecular docking to guide AKR1C3 inhibitor design^19^^,^^35^^,^^63^. In molecular docking, predicted binding poses and binding energies depend on the spatial and physico-chemical properties of the ligand-binding site of the target^64^. Because the active site of AKR1C3 in complex with ibuprofen (PDB 3R8G) is different than that observed in complex with compound 7 (PDB 8RRJ) (Figure 7), the choice of protein “receptor” for docking must be carefully considered^64^^,^^65^. For this purpose, comparative molecular docking was done using structures of AKR1C3 in complex with NSAID inhibitors ibuprofen^20^ (PDB 3R8G) and flufenamic acid^66^ (PDB 1S2C), or compound **7** (PDB 8RRJ) as “receptors” in Autodock Vina. Ibuprofen, flufenamic acid and compound **7** were “redocked” into their native crystal structure or cross-docked against the other two AKR1C3 conformations, and RMSD differences between native ligand poses and docked ligand poses were calculated (see Table 4).

**Table 4.** Comparative molecular docking using superimposed structures of AKR1C3 in complex with NSAID inhibitors ibuprofen (PDB 3R8G) and flufenamic acid (PDB 1S2C) or compound **7** (PDB 8RRJ) as “receptors” in Autodock Vina.

	IC50 AKR1C3		AKR1C3-ibu	AKR1C3-flu	AKR1C3-7
			3R8G	1S2C	8RRJ
Ibuprofen	∼10 µM*				
		RMSD Å	**0.551**	0.817	3.297
		Docking energy (kcal/mol)	**−9.7**	−9.0	−8.6
Flufenamic acid	∼1.7 µM*				
		RMSD Å	1.549	**0.653**	3.464
		Docking energy (kcal/mol)	−9.0	**−10.3**	−9.8
Compound 7	∼14 µM				
		RMSD Å	n.b	n.b	**0.676**
		Docking energy (kcal/mol)	n.b	n.b	**−14.3**

Ibuprofen, flufenamic acid and compound **7** were “redocked” into their native crystal structure or cross-docked against the other two AKR1C3 conformations. RMSD values between the native pose of each ligand in their respective X-ray structure and the docked poses were calculated in Autodock Tools. *Published IC50 values for AKR1C3 inhibition by ibuprofen^20^, flufenamic acid^75^ along with the measured IC50 value for compound **7** are shown for comparison.

**Figure 8.**
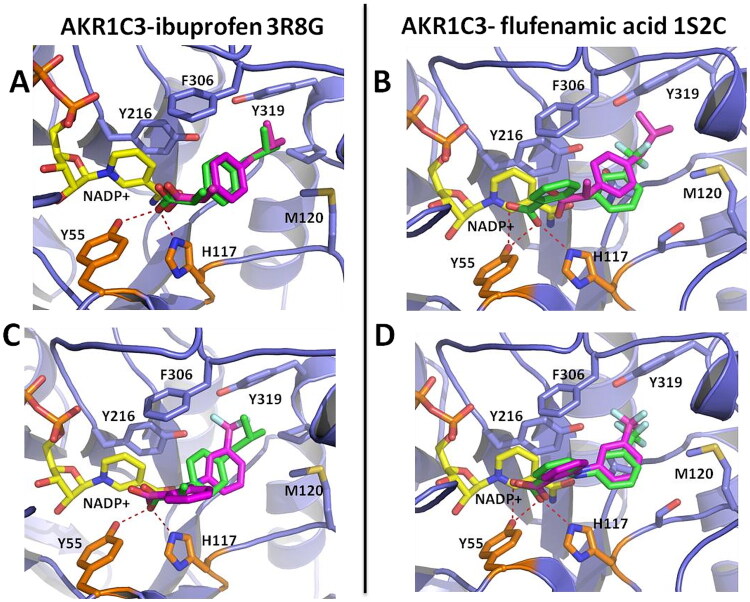
Comparative molecular docking of non-steroidal anti-inflammatory drugs (NSAIDs) using aligned structures of AKR1C3 in complex with inhibitors ibuprofen (PDB 3R8G) or flufenamic acid (PDB 1S2C) as “receptors” in Autodock Vina. Panel A: Redocking of ibuprofen into the native structure of AKR1C3-ibuprofen. The docked pose of ibuprofen (magenta sticks) superimposes onto the experimental binding pose of ibuprofen (green sticks) with an RMSD of 0.551 Å. Panel B: Cross-docking of ibuprofen (magenta sticks) against AKR1C3-flufenamic acid (green sticks). The cross-docked pose of ibuprofen superimposes onto the experimental binding pose of ibuprofen in AKR1C3-ibuprofen with an RMSD of 0.817 Å. Panel C: Cross-docking of flufenamic acid (magenta sticks) against AKR1C3-ibuprofen (green sticks). The cross-docked pose of flufenamic acid superimposes onto the native binding pose of flufenamic acid in AKR1C3-flufenamic acid with an RMSD of 1.549 Å. Panel D: Redocking of flufenamic acid into the native structure of AKR1C3-flufenamic acid. The docked pose of flufenamic acid (magenta sticks) superimposes onto the experimental binding pose of flufenamic acid (green sticks) with an RMSD of 0.653 Å. RMSD values were calculated using Autodock Tools.

Redocking ibuprofen using the active site conformation found in the native AKR1C3-ibuprofen structure (PDB 3R8G) as “receptor” results in an excellent RMSD of 0.551 Å, indicating that Autodock Vina correctly predicts the binding geometry; whereas cross-docking ibuprofen into the active site conformation found in the AKR1C3-flufenamic acid complex (PDB 1S2C) gave a slightly higher RMSD of 0.817 Å (Figure 8). Similarly, redocking flufenamic acid into the active site conformation from the native AKR1C3-flufenamic acid complex (PDB 1S2C) also gave an excellent RMSD of 0.653 Å; while cross-docking flufenamic acid using the AKR1C3-ibuprofen active site conformation (PDB 3R8G) gave a higher RMSD of 1.549 Å. Redocking compound **7** into the active site of the native AKR1C3-7 complex (PDB 8RRJ) also gave an excellent RMSD of 0.676 Å. However, strikingly, cross-docking of compound **7** into either the AKR1C3-ibuprofen (PDB 3R8G) or AKR1C3-flufenamic acid (PDB 1S2C) conformations failed to identify binding in the active site.

Similarly, cross-docking of flufenamic acid or ibuprofen into the active site conformation in our AKR1C3-7 complex (PDB 8RRJ) also failed with much higher RMSD values of 3.464 Å and 3.297 Å, respectively, between native ligand and docked ligand poses. These results are due to conformational differences, such as the position of W227, that depend on ligand-induced changes in the AKR1C3 active site (see Figure 7). In line with this, the highest predicted binding energies for ibuprofen, flufenamic acid and compound **7** were obtained by docking into their native structures, while cross-docking predicted lower binding energies. Note that, although these docking energies are favourable, they do not correlate well with experimental IC50 values. This is expected, since docking energies are obtained via empirical scoring functions designed to rapidly sort libraries of compounds to identify potential ligands. Computationally intensive methods such as free energy perturbation or relative/absolute binding free energy calculations are needed to predict protein-ligand binding free energies *in silico*^67^^,^^68^.

Together our cross-docking results are in agreement with reports that docking predictions of binding poses and energies are more accurate when an experimentally determined structure of the protein target in complex with a ligand that is structurally and chemically similar to the compound of interest is available^64^.

### Molecular docking of compounds 1–8 into the X-ray structure of AKR1C3-7

Because they are chemically and structurally similar to compound **7**, the structural basis of AKR1C3 inhibition by compounds **1–8** was analysed by molecular docking. The X-ray structure of AKR1C3 in complex with compound **7** was used as a “receptor” for molecular docking simulations in the PyRx virtual screening tool using the program Autodock Vina, using a standard docking search space (25 × 25 × 25 Å) centred on the location of compound **7** in the AKR1C3 active site^49^^,^^51^. Because compound **7** interacts with bound waters in the AKR1C3 active site, water molecules were also included in docking simulations. As a positive control, Autodock Vina was able to correctly re-dock compound **7** onto AKR1C3 with a binding geometry identical to the experimental X-ray structure of AKR1C3-7. Compound **7** was also predicted to have the strongest affinity for AKR1C3, with a docking energy −14.3 kcal/mol, in agreement with experimental results. Using these parameters, compounds **1**–**8** were then docked with AKR1C3, and the geometry and binding energy of the top ranking pose for each ligand was analysed. With the exception of compound **5**, docking energies predicted for **1**–**8** by Autodock Vina are in agreement with experimental results from AKR1C3 activity assays *in vitro* (see Table 5). Specifically, based on predicted molecular docking energies, Autodock Vina was able to correctly identify and rank the top three inhibitors (**7**, **1** and **2**) that were shown experimentally to inhibit AKR1C3 activity by ≥60%. Moreover, the weakest AKR1C3 inhibitors (compounds **4** and **6**) were predicted to have the weakest binding affinities by molecular docking (Table 5).

**Table 5.** Compounds **1–8** ranked by predicted Autodock Vina docking energies in comparison with experimental inhibition of AKR1C3 activity.

Compound	%AKR1C3 inhibition	Docking energy (kcal//mol)
**7**	85%	−14.3
**1**	76%	−13.7
**2**	60%	−13.3
**5**	17%	−12.7
**3**	49%	−11.8
**8**	40%	−9.4
**4**	14%	−9.3
**6**	8%	−9.3

**Figure 9.**
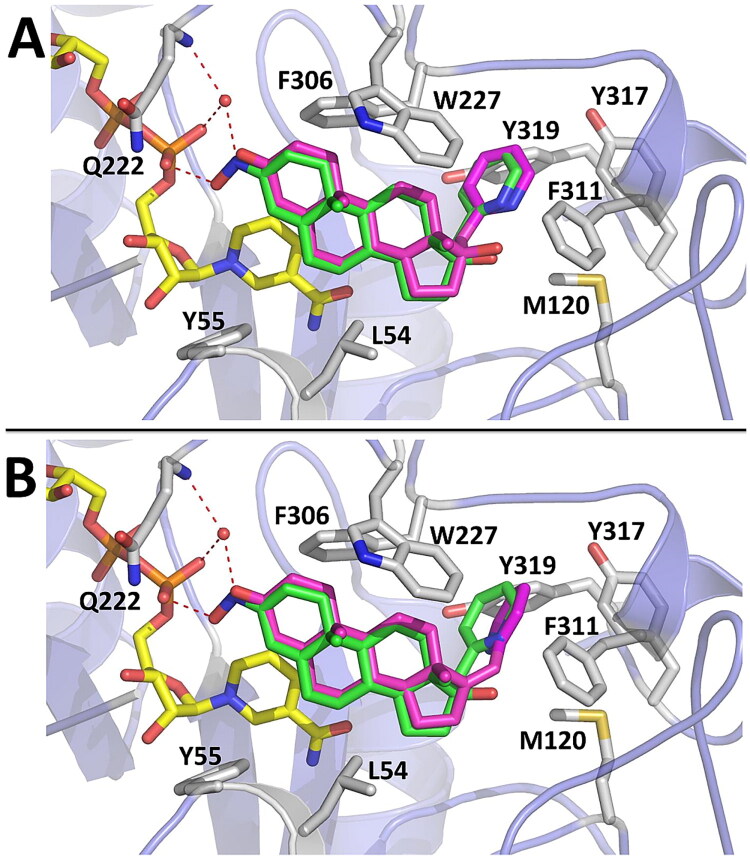
Molecular docking results from Autodock Vina compared with the crystal structure of AKR1C3-7. The top ranking binding geometry predicted by Autodock Vina is shown for compound **1** (panel A) and compound **2** (panel B) in comparison with the X-ray structure of compound **7** (green sticks) in complex with AKR1C3. The NADP^+^ cofactor is shown as yellow sticks. Selected amino acid residues involved in binding to compound **7** are shown as white sticks and labelled. Hydrogen bonds formed by the C3-oxime group of compound **7** are shown as red dashed lines.

Molecular docking was also used to predict and analyse possible binding geometries for top ranking compounds **1** and **2**. As can be seen in Figure 9, compound **1** (76% AKR1C3 inhibition) and **2** (60% AKR1C3 inhibition) were predicted by docking to bind to AKR1C3 with a geometry that is virtually identical to that observed for compound **7** in the X-ray structure. The C3-oxo group present in compounds **1** and **2** could participate in hydrogen bonding to the NADP^+^ cofactor in a manner similar to compound **7**. Docking also predicted that the 17α-picolyl moiety present in compounds **1** and **7** and the 17-picolinylidene moiety present in compound **2** occupy the same site in AKR1C3, via hydrophobic interactions with SP1 (F311 and Y319) and M120.

In agreement with *in vitro* assay results, molecular docking suggests that the structural basis of AKR1C3 inhibition by compounds **7**, **2** and **1** could depend on C3 functional group interactions with the NADP^+^ cofactor and surrounding residues; while the C17 picolyl or picolinylidene appear to anchor the inhibitor in the active site via hydrophobic interactions with residues in SP1.

## Conclusions

In the present study, eight A-ring modified 17α-picolyl and 17(*E*)-picolinylidene androstane derivatives were shown to inhibit reduction of PQ by AKR1C3 *in vitro*. Inhibition of AKR1C3 activity by the tested 17α-picolyl or 17(*E*)-picolinylidene androstane derivatives appears to depend on the identity of the C3 modification: where C3-oxime > C3-oxo > C3-hydroxyl > C3-hydrazone for otherwise identical pairs of compounds. Because compound **2** was also previously shown to inhibit CYP17, results from the present study suggest it may be possible to design dual AKR1C3/CYP17 inhibitors for potential use in the treatment of advanced prostate cancer based on a steroid scaffold. Based on experimental assays, a 17α-picolyl androstane derivative with a C3-oxime modification (compound **7**) was identified as the strongest AKR1C3 inhibitor. The structural basis of AKR1C3 inhibition by compound **7** was determined by X-ray crystallography at 1.7 Å resolution (PDB 8RRJ). In addition, none of the test compounds appear to have androgenic activity, since no binding to the androgen receptor was detected using a fluorescence assay for AR binding conducted in yeast cells. The structural basis of inhibition by the tested 17α-picolyl or 17(*E*)-picolinylidene androstane derivatives was also analysed by molecular docking, where predicted binding energies are in general agreement with results from *in vitro* experimental AKR1C3 assays. Based on molecular docking, *in vitro* assays and X-ray crystallography, the molecular basis of AKR1C3 inhibition by the tested 17α-picolyl or 17(*E*)-picolinylidene androstane derivatives depends on interactions between the C3 modification and the NADP^+^ cofactor, while the C17-picolyl or C17-picolinylidene group anchors the inhibitor through interactions with AKR1C3 subpockets previously shown to be important for inhibitor binding. Combined results from *in vitro* assays, molecular docking and protein X-ray crystallography suggest that A-ring modified 17α-picolyl and 17(E)-picolinylidene androstane derivatives are promising candidates for the design of new AKR1C3 inhibitors.

## Supplementary Material

Supplementary_material.pdf

## Data Availability

Crystallographic data for AKR1C3-NADP^+^ in complex with compound **7** are openly available in the Protein Data Bank (PDB) at https://doi.org/10.2210/pdb8rrj/pdb, reference number 8RRJ.

## References

[1] Byrns MC, Jin Y, Penning TM. Inhibitors of type 5 17β-hydroxysteroid dehydrogenase (AKR1C3): Overview and structural insights. J Steroid Biochem Mol Biol. 2011;125(1–2):95–104. 21087665 10.1016/j.jsbmb.2010.11.004 PMC3047600

[2] Brožič P, Turk S, Rižner TL, Gobec S. Inhibitors of aldo-keto reductases AKR1C1-AKR1C4. Curr Med Chem. 2011;18(17):2554–2565. 21568892 10.2174/092986711795933713

[3] Penning TM. The aldo-keto reductases (AKRs): overview. Chem Biol Interact. 2015;234:236–246. 25304492 10.1016/j.cbi.2014.09.024 PMC4388799

[4] Penning TM, Wangtrakuldee P, Auchus RJ. Structural and functional biology of aldo-keto reductase steroid-transforming enzymes. Endocr Rev. 2019;40(2):447–475. 30137266 10.1210/er.2018-00089 PMC6405412

[5] Yepuru M, Wu Z, Kulkarni A, Yin F, Barrett CM, Kim J, Steiner MS, Miller DD, Dalton JT, Narayanan R, et al. Steroidogenic enzyme AKR1C3 is a novel androgen receptor-selective coactivator that promotes prostate cancer growth. Clin Cancer Res. 2013;19(20):5613–5625. 23995860 10.1158/1078-0432.CCR-13-1151

[6] Liu Y, He S, Chen Y, Liu Y, Feng F, Liu W, Guo Q, Zhao L, Sun H. Overview of AKR1C3: inhibitor achievements and disease insights. J Med Chem. 2020;63(20):11305–11329. 32463235 10.1021/acs.jmedchem.9b02138

[7] Xing S, Jiang J, Chu X, Wang X, Wang Z, Li X, Lv B, Guo C, He S, Wang L, et al. Discovery of highly potent AKR1C3 inhibitors treating sorafenib-resistant hepatocellular carcinoma. J Med Chem. 2025;68(7):7367–7389. 40143712 10.1021/acs.jmedchem.4c03035

[8] Maddeboina K, Jonnalagadda SK, Morsy A, Duan L, Chhonker YS, Murry DJ, Penning TM, Trippier PC. Aldo-keto reductase 1C3 inhibitor prodrug improves pharmacokinetic profile and demonstrates in vivo efficacy in a prostate cancer xenograft model. J Med Chem. 2023;66(14):9894–9915. 37428858 10.1021/acs.jmedchem.3c00732 PMC11963376

[9] Khanim F, Davies N, Veliça P, Hayden R, Ride J, Pararasa C, Chong MG, Gunther U, Veerapen N, Winn P, et al. Selective AKR1C3 inhibitors do not recapitulate the anti-leukaemic activities of the pan-AKR1C inhibitor medroxyprogesterone acetate. Br J Cancer. 2014;110(6):1506–1516. 24569460 10.1038/bjc.2014.83 PMC3960632

[10] Verma K, Zang T, Penning TM, Trippier PC. Potent and highly selective aldo-keto reductase 1C3 (AKR1C3) inhibitors act as chemotherapeutic potentiators in acute myeloid leukemia and T-cell acute lymphoblastic leukemia. J Med Chem. 2019;62(7):3590–3616. 30836001 10.1021/acs.jmedchem.9b00090 PMC6528660

[11] Khanim FL, Hayden RE, Birtwistle J, Lodi A, Tiziani S, Davies NJ, Ride JP, Viant MR, Gunther UL, Mountford JC, et al. Combined bezafibrate and medroxyprogesterone acetate: potential novel therapy for acute myeloid leukaemia. PLoS One. 2009;4(12):e8147. 19997560 10.1371/journal.pone.0008147 PMC2785482

[12] Hertzog JR, Zhang Z, Bignan G, Connolly PJ, Heindl JE, Janetopoulos CJ, Rupnow BA, McDevitt TM. AKR1C3 mediates pan-AR antagonist resistance in castration-resistant prostate cancer. Prostate. 2020;80(14):1223–1232. 33258507 10.1002/pros.24049

[13] Zhao J, Ning S, Lou W, Yang JC, Armstrong CM, Lombard AP, D’Abronzo LS, Evans CP, Gao AC, Liu C, et al. Cross-resistance among next-generation antiandrogen drugs through the AKR1C3/AR-V7 axis in advanced prostate cancer. Mol Cancer Ther. 2020;19(8):1708–1718. 32430485 10.1158/1535-7163.MCT-20-0015 PMC8855880

[14] Heibein AD, Guo B, Sprowl JA, MacLean DA, Parissenti AM. Role of aldo-keto reductases and other doxorubicin pharmacokinetic genes in doxorubicin resistance, DNA binding, and subcellular localization. BMC Cancer. 2012;12(1):381. 22938713 10.1186/1471-2407-12-381 PMC3495881

[15] Liu C, Armstrong CM, Lou W, Lombard A, Evans CP, Gao AC. Inhibition of AKR1C3 activation overcomes resistance to abiraterone in advanced prostate cancer. Mol Cancer Ther. 2017;16(1):35–44. 27794047 10.1158/1535-7163.MCT-16-0186 PMC5222693

[16] Shiiba M, Yamagami H, Yamamoto A, Minakawa Y, Okamoto A, Kasamatsu A, Sakamoto Y, Uzawa K, Takiguchi Y, Tanzawa H, et al. Mefenamic acid enhances anticancer drug sensitivity via inhibition of aldo-keto reductase 1C enzyme activity. Oncol Rep. 2017;37(4):2025–2032. 28259989 10.3892/or.2017.5480

[17] Bauman DR, Rudnick SI, Szewczuk LM, Jin Y, Gopishetty S, Penning TM. Development of nonsteroidal anti-inflammatory drug analogs and steroid carboxylates selective for human aldo-keto reductase isoforms: potential antineoplastic agents that work independently of cyclooxygenase isozymes. Mol Pharmacol. 2005;67(1):60–68. 15475569 10.1124/mol.104.006569

[18] Brožič P, Turk S, Adeniji AO, Konc J, Janežič D, Penning TM, Lanišnik Rižner T, Gobec S. Selective inhibitors of aldo-keto reductases AKR1C1 and AKR1C3 discovered by virtual screening of a fragment library. J Med Chem. 2012;55(17):7417–7424. 22881866 10.1021/jm300841n PMC3470935

[19] Savić MP, Ajduković JJ, Plavša JJ, Bekić SS, Ćelić AS, Klisurić OR, Jakimov DS, Petri ET, Djurendić EA. Evaluation of A-ring fused pyridine d-modified androstane derivatives for antiproliferative and aldo-keto reductase 1C3 inhibitory activity. Medchemcomm. 2018;9(6):969–981. 30108986 10.1039/c8md00077h PMC6071935

[20] Flanagan JU, Yosaatmadja Y, Teague RM, Chai MZL, Turnbull AP, Squire CJ. Crystal structures of three classes of non-steroidal anti-inflammatory drugs in complex with aldo-keto reductase 1C3. PLoS One. 2012;7(8):e43965. 22937138 10.1371/journal.pone.0043965 PMC3429426

[21] Rebello RJ, Oing C, Knudsen KE, Loeb S, Johnson DC, Reiter RE, Gillessen S, Van der Kwast T, Bristow RG. Prostate cancer. Nat Rev Dis Primers. 2021;7(1):9. 33542230 10.1038/s41572-020-00243-0

[22] Dai C, Heemers H, Sharifi N. Androgen signaling in prostate cancer. Cold Spring Harb Perspect Med. 2017;7(9):a030452. 28389515 10.1101/cshperspect.a030452 PMC5580512

[23] Ajduković JJ, Djurendić EA, Petri ET, Klisurić OR, Celić AS, Sakač MN, Jakimov DS, Gaši KMP. 17(E)-picolinylidene androstane derivatives as potential inhibitors of prostate cancer cell growth: antiproliferative activity and molecular docking studies. Bioorg Med Chem. 2013;21(23):7257–7266. 24148837 10.1016/j.bmc.2013.09.063

[24] Szabó N, Ajduković JJ, Djurendić EA, Sakač MN, Ignáth I, Gardi J, Mahmoud G, Klisurić OR, Jovanović-Šanta S, Penov Gaši KM, et al. Determination of 17α-hydroxylase-C17,20-lyase (P45017α) enzyme activities and their inhibition by selected steroidal picolyl and picolinylidene compounds. Acta Biol Hung. 2015;66(1):41–51. 25740437 10.1556/ABiol.66.2015.1.4

[25] Harshman LC, Taplin M-E. Abiraterone acetate: targeting persistent androgen dependence in castration-resistant prostate cancer. Adv Ther. 2013;30(8):727–747. 23979793 10.1007/s12325-013-0050-3 PMC3778906

[26] Swami U, McFarland TR, Nussenzveig R, Agarwal N. Advanced prostate cancer: treatment advances and future directions. Trends Cancer. 2020;6(8):702–715. 32534790 10.1016/j.trecan.2020.04.010

[27] Mostaghel EA, Marck BT, Plymate SR, Vessella RL, Balk S, Matsumoto AM, Nelson PS, Montgomery RB. Resistance to CYP17A1 inhibition with abiraterone in castration-resistant prostate cancer: induction of steroidogenesis and androgen receptor splice variants. Clin Cancer Res. 2011;17(18):5913–5925. 21807635 10.1158/1078-0432.CCR-11-0728 PMC3184252

[28] Liu C, Lou W, Zhu Y, Yang JC, Nadiminty N, Gaikwad NW, Evans CP, Gao AC. Intracrine androgens and AKR1C3 activation confer resistance to enzalutamide in prostate cancer. Cancer Res. 2015;75(7):1413–1422. 25649766 10.1158/0008-5472.CAN-14-3080 PMC4383695

[29] Kafka M, Mayr F, Temml V, Möller G, Adamski J, Höfer J, Schwaiger S, Heidegger I, Matuszczak B, Schuster D, et al. Dual inhibitory action of a novel AKR1C3 inhibitor on both full-length AR and the variant AR-V7 in enzalutamide resistant metastatic castration resistant prostate cancer. Cancers (Basel). 2020;12(8):2092. 32731472 10.3390/cancers12082092 PMC7465893

[30] Armstrong CM, Gao AC. Dysregulated androgen synthesis and anti-androgen resistance in advanced prostate cancer. Am J Clin Exp Urol. 2021;9(4):292–300. 34541028 PMC8446765

[31] Liedtke AJ, Adeniji AO, Chen M, Byrns MC, Jin Y, Christianson DW, Marnett LJ, Penning TM. Development of potent and selective indomethacin analogues for the inhibition of AKR1C3 (Type 5 17β-hydroxysteroid dehydrogenase/prostaglandin F synthase) in castrate-resistant prostate cancer. J Med Chem. 2013;56(6):2429–2446. 23432095 10.1021/jm3017656 PMC3638264

[32] Veitch ZW, Guo B, Hembruff SL, Bewick AJ, Heibein AD, Eng J, Cull S, Maclean DA, Parissenti AM. Induction of 1C aldoketoreductases and other drug dose-dependent genes upon acquisition of anthracycline resistance. Pharmacogenet Genomics. 2009;19(6):477–488. 19440163 10.1097/FPC.0b013e32832c484b

[33] Djurendić E, Daljev J, Sakac M, Canadi J, Santa SJ, Andrić S, Klisurić O, Kojić V, Bogdanović G, Djurendić-Brenesel M, et al. Synthesis of some epoxy and/or N-oxy 17-picolyl and 17-picolinylidene-androst-5-ene derivatives and evaluation of their biological activity. Steroids. 2008;73(1):129–138. 17963806 10.1016/j.steroids.2007.09.005

[34] Ajduković JJ, Jakimov DS, Rárová L, Strnad M, Dzichenka YU, Usanov S, Škorić DĐ, Jovanović-Šanta SS, Sakač MN. Novel alkylaminoethyl derivatives of androstane 3-oximes as anticancer candidates: synthesis and evaluation of cytotoxic effects. RSC Adv. 2021;11(59):37449–37461. 35496404 10.1039/d1ra07613b PMC9043769

[35] Marinović MA, Petri ET, Grbović LM, Vasiljević BR, Jovanović-Šanta SS, Bekić SS, Ćelić AS. Investigation of the potential of bile acid methyl esters as inhibitors of aldo-keto reductase 1C2: insight from molecular docking, virtual screening, experimental assays and molecular dynamics. Mol Inform. 2022;41(10):e2100256. 35393780 10.1002/minf.202100256

[36] Plavša JJ, Řezáčová P, Kugler M, Pachl P, Brynda J, Voburka Z, Ćelić A, Petri ET, Škerlová J. In situ proteolysis of an N-terminal His tag with thrombin improves the diffraction quality of human aldo-keto reductase 1C3 crystals. Acta Crystallogr F Struct Biol Commun. 2018;74(Pt 5):300–306. 29717998 10.1107/S2053230X18005721 PMC5931143

[37] Gorrec F. The MORPHEUS protein crystallization screen. J Appl Crystallogr. 2009;42(Pt 6):1035–1042. 22477774 10.1107/S0021889809042022 PMC3246824

[38] Mueller U, Darowski N, Fuchs MR, Förster R, Hellmig M, Paithankar KS, Pühringer S, Steffien M, Zocher G, Weiss MS, et al. Facilities for macromolecular crystallography at the Helmholtz-Zentrum Berlin. J Synchrotron Radiat. 2012;19(Pt 3):442–449. 22514183 10.1107/S0909049512006395 PMC3408958

[39] Gerlach M, Mueller U, Weiss MS. The MX beamlines BL14. 1-3 at BESSY II. JLSRF. 2016;2:A47.

[40] Kabsch W. XDS. Acta Crystallogr D Biol Crystallogr. 2010;66(Pt 2):125–132. 20124692 10.1107/S0907444909047337 PMC2815665

[41] McCoy AJ, Grosse-Kunstleve RW, Adams PD, Winn MD, Storoni LC, Read RJ. Phaser crystallographic software. J Appl Crystallogr. 2007;40(Pt 4):658–674. 19461840 10.1107/S0021889807021206 PMC2483472

[42] Qiu W, Zhou M, Mazumdar M, Azzi A, Ghanmi D, Luu-The V, Labrie F, Lin S-X. Structure-based inhibitor design for an enzyme that binds different steroids: a potent inhibitor for human type 5 17beta-hydroxysteroid dehydrogenase. J Biol Chem. 2007;282(11):8368–8379. 17166832 10.1074/jbc.M606784200

[43] Winn MD, Ballard CC, Cowtan KD, Dodson EJ, Emsley P, Evans PR, Keegan RM, Krissinel EB, Leslie AGW, McCoy A, et al. Overview of the CCP4 suite and current developments. Acta Crystallogr D Biol Crystallogr. 2011;67(Pt 4):235–242. 21460441 10.1107/S0907444910045749 PMC3069738

[44] Murshudov GN, Vagin AA, Dodson EJ. Refinement of macromolecular structures by the maximum-likelihood method. Acta Crystallogr D Biol Crystallogr. 1997;53(Pt 3):240–255. 15299926 10.1107/S0907444996012255

[45] Emsley P, Lohkamp B, Scott WG, Cowtan K. Features and development of Coot. Acta Crystallogr D Biol Crystallogr. 2010;66(Pt 4):486–501. 20383002 10.1107/S0907444910007493 PMC2852313

[46] Winn MD, Murshudov GN, Papiz MZ. Macromolecular TLS refinement in REFMAC at moderate resolutions. In: Macromolecular crystallography, part D. Vol 374. Methods in Enzymology. Cambridge, MA: Academic Press; 2003: p. 300–321. 10.1016/S0076-6879(03)74014-2 14696379

[47] Pedretti A, Villa L, Vistoli G. Atom-type description language: a universal language to recognize atom types implemented in the VEGA program. Theor Chem Acc. 2003;109(4):229–232.

[48] Pedretti A, Mazzolari A, Gervasoni S, Fumagalli L, Vistoli G. The VEGA suite of programs: an versatile platform for cheminformatics and drug design projects. Bioinformatics. 2021;37(8):1174–1175. 33289523 10.1093/bioinformatics/btaa774

[49] Trott O, Olson AJ. AutoDock Vina: improving the speed and accuracy of docking with a new scoring function, efficient optimization, and multithreading. J Comput Chem. 2010;31(2):455–461. 19499576 10.1002/jcc.21334 PMC3041641

[50] Hanwell MD, Curtis DE, Lonie DC, Vandermeersch T, Zurek E, Hutchison GR. Avogadro: An advanced semantic chemical editor, visualization, and analysis platform. J Cheminform. 2012;4(1):17. 22889332 10.1186/1758-2946-4-17 PMC3542060

[51] Dallakyan S, Olson AJ. Small-molecule library screening by docking with PyRx. In: Methods in molecular biology. Vol 1263. New York: Springer; 2015: p. 243–250. 25618350 10.1007/978-1-4939-2269-7_19

[52] DeLano WL. The case for open-source software in drug discovery. Drug Discov Today. 2005;10(3):213–217. 15708536 10.1016/S1359-6446(04)03363-X

[53] Muddana SS, Peterson BR. Fluorescent cellular sensors of steroid receptor ligands. Chembiochem. 2003;4(9):848–855. 12964159 10.1002/cbic.200300606

[54] Gietz D, Jean AS, Woods RA, Schiestl RH. Improved method for high efficiency transformation of intact yeast cells. Nucleic Acids Res. 1992;20(6):1425–1425. 1561104 10.1093/nar/20.6.1425 PMC312198

[55] Brozic P, Smuc T, Gobec S, Rizner TL. Phytoestrogens as inhibitors of the human progesterone metabolizing enzyme AKR1C1. Mol Cell Endocrinol. 2006;259(1–2):30–42. 16962702 10.1016/j.mce.2006.08.001

[56] Byrns MC, Steckelbroeck S, Penning TM. An indomethacin analogue, N-(4-chlorobenzoyl)-melatonin, is a selective inhibitor of aldo-keto reductase 1C3 (type 2 3alpha-HSD, type 5 17beta-HSD, and prostaglandin F synthase), a potential target for the treatment of hormone dependent and hormone indep. Biochem Pharmacol. 2008;75(2):484–493. 17950253 10.1016/j.bcp.2007.09.008 PMC2245880

[57] Adeniji AO, Chen M, Penning TM. AKR1C3 as a target in castrate resistant prostate cancer. J Steroid Biochem Mol Biol. 2013;137:136–149. 23748150 10.1016/j.jsbmb.2013.05.012 PMC3805777

[58] Bekić SS, Marinović MA, Petri ET, Sakač MN, Nikolić AR, Kojić VV, Ćelić AS. Identification of D-seco modified steroid derivatives with affinity for estrogen receptor α and β isoforms using a non-transcriptional fluorescent cell assay in yeast. Steroids. 2018;130:22–30. 29224741 10.1016/j.steroids.2017.12.002

[59] Vasiljević BR, Petri ET, Bekić SS, Ćelić AS, Grbović LM, Pavlović KJ. Microwave-assisted green synthesis of bile acid derivatives and evaluation of glucocorticoid receptor binding. RSC Med Chem. 2021;12(2):278–287. 34046616 10.1039/d0md00311e PMC8128055

[60] Endo S, Matsunaga T, Kanamori A, Otsuji Y, Nagai H, Sundaram K, El-Kabbani O, Toyooka N, Ohta S, Hara A, et al. Selective inhibition of human type-5 17β-hydroxysteroid dehydrogenase (AKR1C3) by baccharin, a component of Brazilian propolis. J Nat Prod. 2012;75(4):716–721. 22506594 10.1021/np201002x

[61] Laskowski RA, Swindells MB. LigPlot+: multiple ligand-protein interaction diagrams for drug discovery. J Chem Inf Model. 2011;51(10):2778–2786. 21919503 10.1021/ci200227u

[62] Wallace AC, Laskowski RA, Thornton JM. LIGPLOT: a program to generate schematic diagrams of protein-ligand interactions. Protein Eng. 1995;8(2):127–134. 7630882 10.1093/protein/8.2.127

[63] Marinović MA, Bekić SS, Kugler M, Brynda J, Škerlová J, Škorić DĐ, Řezáčová P, Petri ET, Ćelić AS. X-ray structure of human aldo-keto reductase 1C3 in complex with a bile acid fused tetrazole inhibitor: experimental validation, molecular docking and structural analysis. RSC Med Chem. 2023;14(2):341–355. 36846371 10.1039/d2md00387b PMC9945864

[64] Paggi JM, Pandit A, Dror RO. The art and science of molecular docking. Annu Rev Biochem. 2024;93(1):389–410. 38594926 10.1146/annurev-biochem-030222-120000 PMC13198409

[65] Saikia S, Bordoloi M. Molecular docking: challenges, advances and its use in drug discovery perspective. Curr Drug Targets. 2019;20(5):501–521. 30360733 10.2174/1389450119666181022153016

[66] Lovering AL, Ride JP, Bunce CM, Desmond JC, Cummings SM, White SA. Crystal structures of prostaglandin D2 11-ketoreductase (AKR1C3) in complex with the nonsteroidal anti-inflammatory drugs flufenamic acid and indomethacin. Cancer Res. 2004;64(5):1802–1810. 14996743 10.1158/0008-5472.can-03-2847

[67] Jespers W, Åqvist J, Gutiérrez-de-Terán H. Free energy calculations for protein-ligand binding prediction. Methods Mol Biol. 2021;2266:203–226. 33759129 10.1007/978-1-0716-1209-5_12

[68] Fu H, Chipot C, Shao X, Cai W. Standard binding free-energy calculations: how far are we from automation? J Phys Chem B. 2023;127(49):10459–10468. 37824848 10.1021/acs.jpcb.3c04370

[69] Gaillard P, Carrupt PA, Testa B, Boudon A. Molecular lipophilicity potential, a tool in 3D QSAR: method and applications. J Comput Aided Mol Des. 1994;8(2):83–96. 7914913 10.1007/BF00119860

[70] Diederichs K, Karplus PA. Improved R-factors for diffraction data analysis in macromolecular crystallography. Nat Struct Biol. 1997;4(4):269–275. 9095194 10.1038/nsb0497-269

[71] Karplus PA, Diederichs K. Linking crystallographic model and data quality. Science. 2012;336(6084):1030–1033. 22628654 10.1126/science.1218231 PMC3457925

[72] The CCP4 suite: programs for protein crystallography. Acta Crystallogr D Biol Crystallogr. 1994;50(Pt 5):760–763. 15299374 10.1107/S0907444994003112

[73] Brünger AT. Free R value: a novel statistical quantity for assessing the accuracy of crystal structures. Nature. 1992;355(6359):472–475. 18481394 10.1038/355472a0

[74] Chen VB, Arendall WB, Headd JJ, Keedy DA, Immormino RM, Kapral GJ, Murray LW, Richardson JS, Richardson DC. MolProbity: all-atom structure validation for macromolecular crystallography. Acta Crystallogr D Biol Crystallogr. 2010;66(Pt 1):12–21. 20057044 10.1107/S0907444909042073 PMC2803126

[75] Chen M, Adeniji AO, Twenter BM, Winkler JD, Christianson DW, Penning TM. Crystal structures of AKR1C3 containing an N-(aryl)amino-benzoate inhibitor and a bifunctional AKR1C3 inhibitor and androgen receptor antagonist. Therapeutic leads for castrate resistant prostate cancer. Bioorg Med Chem Lett. 2012;22(10):3492–3497. 22507964 10.1016/j.bmcl.2012.03.085 PMC3348334

