# Supplementary material for: The structural basis of aldo-keto reductase 1C3 inhibition by 17α-picolyl and 17(E)-picolinylidene androstane derivatives
Source: J Enzyme Inhib Med Chem. 2025 Sep 4;40(1):2551979. doi: 10.1080/14756366.2025.2551979 (PMC12412325; doi:10.1080/14756366.2025.2551979)
Supplement: Supplementary_material.pdf [file IENZ_A_2551979_SM1971.pdf]

The Structural Basis of Aldo-keto Reductase 1C3 Inhibition by 17α-picolyl and 17(*E*)-picolinylidene Androstane Derivatives

Supplementary material

Chemical characterization data for compounds 1-8

17β-hydroxy-17α-picolyl-androst-4-en-3-one (1): mp 184–185 °C. IR (KBr, ν, cm^{-1}): 3423, 1673, 1616, 1596, 1569, 1052,1024, 765, 513. ^{1}H NMR ($CDCl_3$, δ, ppm): 1.00 (3H, s, H-18); 1.21 (3H, s, H-19); 2.79 (1H, d, J_{gem} = 14.6 Hz, CH_2-Py); 3.06 (1H, d, J_{gem} = 14.6 Hz, CH_2-Py); 5.74 (1H, s, H-4); 6.70 (1H, bs, OH-17); 7.15 (d, 1H, *J* = 7.7 Hz, H-3', Py), 7.17 (m, 1H, H-5', Py), 7.63 (td, 1H, J_1 = 7.7 Hz, J_2 = 1.8 Hz, H-4', Py); 8.47 (m, 1H, H-6', Py). ^{13}C NMR ($CDCl_3$, δ, ppm): 199.7 (C-3); 171.5 (C-5); 160.6 (C-2', Py); 147.9 (C-6', Py); 136.8 (C-4', Py); 124.7 (C-3', Py); 123.8 (C-4); 121.4 (C-5', Py); 83.3 (C-17); 53.9; 50.1; 46.4; 43.0; 38.7; 36.3; 35.9; 35.7; 34.0; 32.9; 32.0; 31.8; 23.8; 20.7; 17.4; 14.1. MS: 286 (100;M^{+}-CH_2Py); 244 (55); 124 (53). Anal. calcd. for $C_{25}H_{33}NO_2$ 0.5 H_2O: C, 77.32; H, 8.76; N, 3.61; found: C, 77.62; H, 8.50; N, 3.49.

17-picolinylidene-androst-4-en-3-one (2): mp 166–167 °C. IR (KBr, ν, cm^{-1}): 1673, 1652, 1614, 1581, 1559, 864, 782. ^{1}H NMR ($CDCl_3$, δ, ppm): 0.95 (3H, s, H-18); 1.23 (3H, s, H-19); 2.60-3.00 (2H, m, H-16a and H-16b); 5.75 (1H, s, H-4); 6.23 (1H, s, H-20); 7.04, 7.28, 7.61, 8.57 (4H, Py). ^{13}C NMR ($CDCl_3$, δ, ppm): 199.49 (C-3); 171,12; 159.74; 157.43; 149.14; 135.85; 123.92; 122.80; 120.27; 118.13; 54.02 (CH); 53.09 (CH); 45.71; 38.69; 35.71; 35.65; 35.45; 33.95; 32.83; 31.83; 29.75; 24.90; 21.08; 18.77(C-19); 17.40(C-18). MS: 361 (100; M+); 342 (42). Anal. calcd. for $C_{25}H_{31}NO$ 0.5 H_2O: C, 81.08; H, 8.65; found: C, 81.64; H, 8.63.

3 β,17β -dihydroxy-17α-picolyl-androst-5-ene (3): mp 176-178 °C. IR (KBr, ν, cm^{-1}): 3318, 2930, 2867, 1661, 1598, 1568, 1477, 1435, 1377, 1102, 1061, 1033, 1004, 753. ^{1}H NMR ($CDCl_3$, δ, ppm): 0.96 (s, 3H, H-18); 1.04 (s, 3H, H-19); 2.80 (d, 1H, J_{gem} = 14.5 Hz, C$\underline{H}_2$Py); 3.09 (d, 1H, J_{gem} = 14.5 Hz, CH_2-Py); 3.53 (m, 1H, H-3); 5.36 (d, 1H, *J* = 5.0 Hz, H-6); 7.16; 7.64 i 8.46 (m, 4H, Py). ^{13}C NMR ($CDCl_3$, δ, ppm): 14.11 (C-18); 19.40 (C-19); 20.79; 23.97; 31.67; 31.85; 32.19; 32.75; 35.96; 36.64; 37.37; 42.29; 43.11; 46.37; 50.28; 51.14; 71.77 (C-3); 83.53 (C-17); 121.39 (C-6 and C-5’, Py); 124.86 (C-3’, Py); 136.91 (C-4’, Py); 140.92 (C-5); 147.80 (C-6’, Py); 160.74 (C-2’, Py).

3 β -hydroxy-17-picolinylidene-androst-5-ene (4): mp 175 °C. IR (KBr, ν, cm^{-1}): 3220, 3070, 1650, 1590, 1570, 1470, 1440, 1370, 1350, 1080, 780. ^{1}H NMR ($CDCl_3$, δ, ppm): 0.91 (3H, s, H-18); 1.05 (3H, s, H-19); 2.73 (1H, m, H-16a); 2.89 (1H, m, H-16b); 3.55 (1H, m, H-3); 5.36 (1H, m, H-6); 6.24 (1H, t, $J_{20,16a}$ = $J_{20,16b}$ = 2.4 Hz, H-20); 7.04, 7.30, 7.62, and 8.56 (4H, Py). ^{13}C NMR ($CDCl_3$, δ, ppm): 160.4 (qC); 157.6 (qC); 149.2 (CH); 140.9 (qC); 135.8 (CH); 122.8 (CH); 121.4 (CH); 120.2 (CH); 118.0 (CH); 71.7 (C-3); 54.0 (CH); 50.4 (CH); 45.7 (C-13); 42.3; 37.3; 36.6 (C-10); 35.8; 31.8; 31.7; 31.6; 29.8; 25.1; 21.1; 19.5 (CH_3); 18.6 (CH_3). MS: 363 (100; M+); 348 (46). Anal. calcd. for $C_{25}H_{33}NO$: C, 82.60; H, 9.15; N, 3.85; found: C, 82.70; H, 9.12; N, 3.90.

17β -Hydroxy-17α-picolyl-androst-4-en-3-on hydrazone (5): mp > 260 °C. IR (KBr, ν, cm^{-1}): 3334, 2938, 2856, 1671, 1629, 1596, 1473, 1437, 1378, 1330, 1240, 1125, 1023, 873, 755; ^{1}H NMR ($CDCl_3$, δ, ppm) 1.00 (3H, s, H-18), 1.11 (3H, s, H-19), 2.79 (1H, d, J_{gem} = 14.7 Hz, CH2Py), 3.06 (1H, d, J_{gem} = 14.7 Hz, CH_2Py), 5.98 (1H, s, H-4), 6.67 (2H, bs, NH_2), 7.16 (2H, m, H-30 and H-5', Py), 7.63 (1H, td, $J_{4',3'}$ = $J_{4',5'}$ = 7.7 Hz, $J_{4',6'}$ = 1.3 Hz, H-4', Py), 8.46 (1H, m, H-6', Py); ^{13}C NMR ($CDCl_3$, δ, ppm) 14.14 (C-18), 17.74 (C-19), 21.03, 21.92, 23.89, 32.19, 32.68, 35.46, 35.96, 36.50, 38.30, 39.03, 43.13, 46.38, 50.31, 53.98, 83.37 (C-17), 113.81 (C4), 121.34 (C-5', Py), 124.73 (C-3', Py), 136.76 (C-4', Py), 147.99 (C-6', Py), 158.31 (C-3), 160.79 (C-2', Py), 161.40 (C-5). MS (ESI), m/z: 380.25 (100), 252.51 (28), 223.66 (14).

17(E)-Picolinylidene-androst-4-en-3-on hydrazone (6): mp 257-259 °C. IR (KBr, ν, cm^{-1}): 2940, 2852, 1653, 1625, 1585, 1560, 1470, 1428, 1373, 1259, 1237, 1215, 1150, 1103, 885, 754; ^{1}H NMR ($CDCl_3$, δ, ppm) 0.93 (6H, 2s, H-18 and H-19), 5.99 (1H, s, H-4), 6.22 (2H, s, NH_2), 6.45 (1H, s, H-20), 7.04 (1H, m, H-5', Py), 7.28 (1H, m, H-3', Py), 7.61 (1H, td, $J_{4',3'}$ = $J_{4',5'}$ = 7.7 Hz, $J_{4',6'}$ = 1.8 Hz, H-4', Py), 8.56 (1H, d, $J_{6',5'}$= 4.8 Hz, H-6', Py); ^{13}C NMR ($CDCl_3$, δ, ppm) 17.93 (C-18), 18.79 (C-19), 21.36, 21.89, 24.95, 27.96, 29.78, 32.27, 35.40, 35.56, 38.27, 38.99, 45.76, 53.25, 54.01, 113.93 (C-4), 118.01 (C-20), 120.17 (C-5', Py), 122.76 (C-3', Py), 135.77 (C-4', Py), 149.14 (C-6', Py), 157.55 (C-17), 158.73 (C-3), 160.16 (C-2', Py), 161.57 (C-5). MS (ESI), m/z: 376.25 (6, $(M+H)^+$), 360.25 (28), 251.17 (25), 240.51 (100).

17 β -Hydroxy-17α-picolyl-androst-4-en-(3Z)-one oxime (7): IR (film, ν, cm^{-1}): 3267, 2940, 1631, 1597, 1570, 1474, 1437, 1024, 999, 971, 944, 910, 754, 665; ^{1}H NMR (DMSO-d6, δ, ppm) 0.85 (s, 3H, H-18), 1.08 (s, 3H, H-19), 2.77 (d, 1H, J = 13.6 Hz, CH_2Py), 2.87 (d, 1H, J = 13.6 Hz, CH_2Py), 5.29 (s, 1H, 17β-OH), 6.34 (s, 1H, H-4), 7.23 (m, 1H, H-3', Py), 7.38 (m, 1H, H-5', Py), 7.69 (m, 1H, H-4', Py), 8.46 (d, 1H, J = 4.8 Hz, H-6', Py), 10.22 (s, 1H, =NOH); ^{13}C NMR (DMSO-d6, δ, ppm) 14.83 (C-18), 18.29 (C-19), 21.04, 23.95, 24.79, 31.68, 32.60, 32.82, 33.99, 36.58, 36.62, 38.94, 43.82, 46.65, 49.83, 54.01, 82.93 (C-17), 111.78 (C-4), 121.78 (C-5', Py), 125.90 (C-3', Py), 136.77 (C-4' , Py), 148.40 (C-6', Py), 151.47 (C-3), 156.98 (C-5), 160.77 (C-2', Py); HRMS (m/z): for $C_{25}H_{35}N_2O_2$ $[M + H]^+$ calcd 395.26985, found 395.26968.

(17E)-Picolinylidene-androst-4-en-(3E)-one oxime (8): IR (film, ν, cm^{-1}): 3186, 3052, 2940, 2878, 2854, 1632, 1591, 1469, 1436, 1373, 1240, 1216, 971, 941, 909, 874, 754, 666. ^{1}H NMR (DMSO-d6, δ, ppm) 0.88 (s, 3H, H-18), 1.06 (s, 3H, H-19), 2.74–2.90 (m, 2H, H-16a and H-16b), 5.72 (s, 1H, H-4), 6.15 (s, 1H, H-20), 7.12 (m, 1H, H-5', Py), 7.31 (m, 1H, H-3', Py), 7.70 (td, 1H, J_1=7.8 Hz, J_2=1.9 Hz, H-4', Py), 8.52 (m, 1H, H-6', Py), 10.47 (s, 1H, =NOH); ^{13}C NMR (DMSO-d6, δ, ppm) 18.00 (C-18), 18.97, 19.16 (C-19), 21.49, 25.00, 30.35, 32.13, 32.28, 34.86, 35.55, 35.90, 38.00, 45.73, 53.43, 53.93, 117.63 (C20), 118.44 (C-4), 120.84 (C-5', Py), 123.48 (C-3', Py), 136.61 (C-4', Py), 149.44 (C-6', Py), 152.98 (C-3), 154.58 (C-5), 157.38 (C-17), 160.37 (C-2', Py); HRMS (m/z): for $C_{25}H_{33}N_2O$ $[M + H]^+$ calcd 377.25929, found 377.25960.

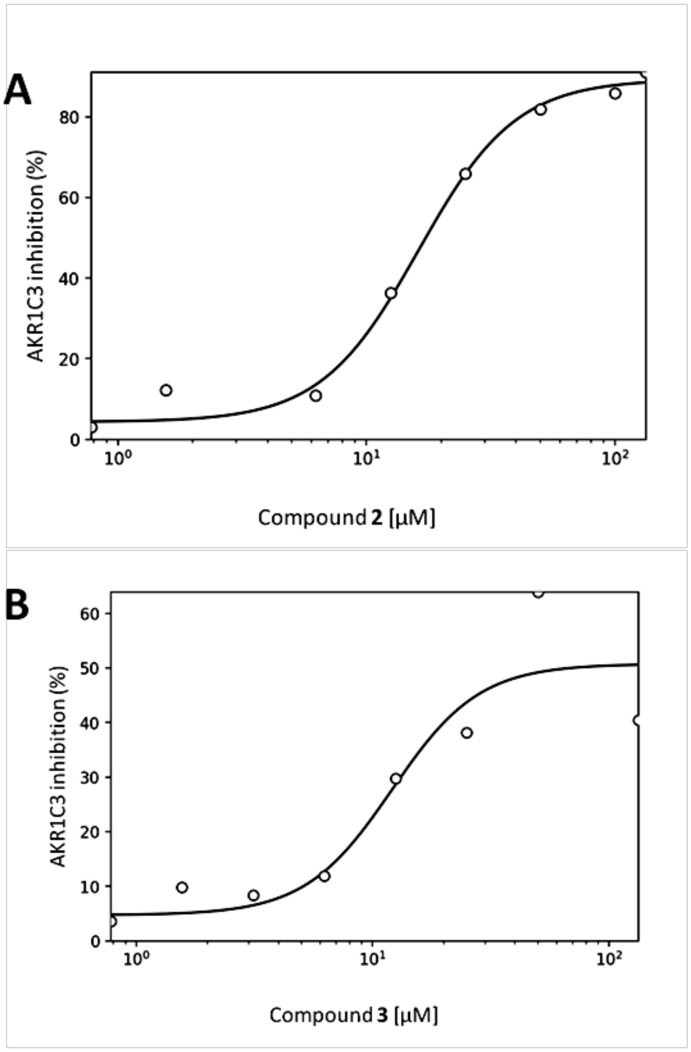

Figure S1: Dose-response curves for inhibition of AKR1C3 upon treatment with increasing concentrations of compound **2** (panel A) or compound **3** (panel B). Percent inhibition of AKR1C3 activity was measured by monitoring changes in NADPH fluorescence over time during reduction of 9,10-phenanthrenequinone following treatment with 0, 0.78, 1.56, 3.12, 6.25, 12.5, 25, 50, 100 and 133 µM of compound **2** or **3**. Reactions contained human AKR1C3 (80 µg/mL), 250 µM NADPH and 4 µM PQ in 100 mM potassium phosphate buffer pH 6.0. Enzyme and compounds were preincubated for 15 minutes at 37 °C and reaction was initiated by addition of NADPH and substrate. Fluorescence was measured in kinetic mode every 30 seconds for 10 minutes at 37 °C using excitation/emission wavelengths of 340/460 nm in a Fluoroskan Ascent FL fluorimeter. Enzyme activity was expressed relative to uninhibited control (defined as 100% activity) based on the slope of fluorescence vs. time data. IC50 values were determined using an online tool https://ic50.org/, yielding calculated IC50 values of 16.17 ± 1.52 µM for compound **2** and 12.09 ± 4.16 µM for compound **3**.

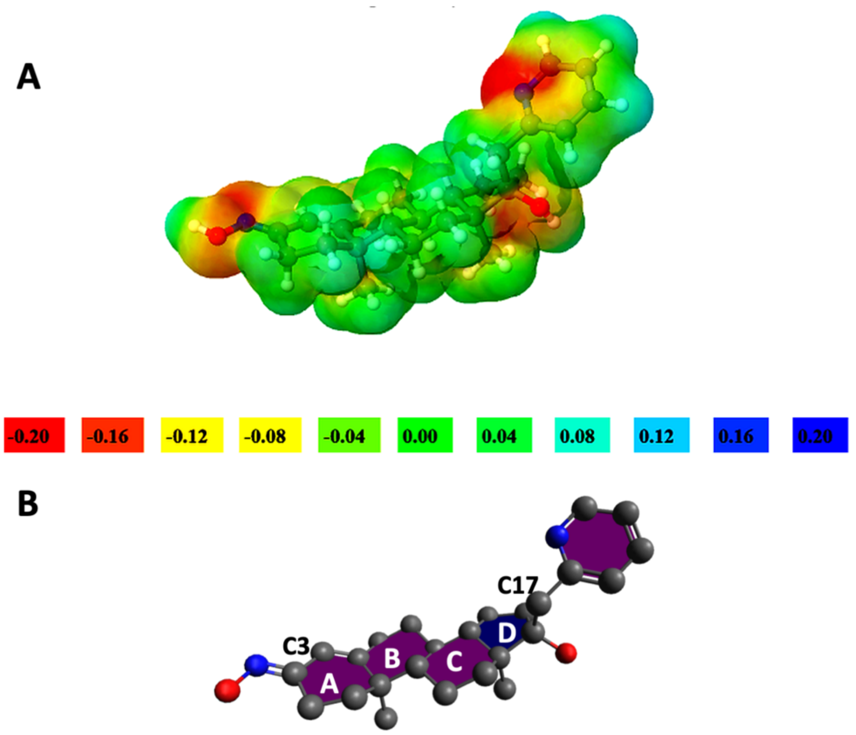

Figure S2: A) Calculated molecular electrostatic potential (MEP) map of compound **7**. The MEP of compound **7** was calculated from the structure of compound **7** using the GAMESS (General Atomic and Molecular Electronic Structure System) ab initio quantum chemistry package at the RHF/3-21G level of theory.[1] MEP partial charges are colored according to the gradient shown. Calculations and visualization were conducted using the Chem Compute Science Gateway resources at chemcompute.org[2] and JSmol viewer.[3] B) To help visualize the MEP map of compound **7**, a simplified ball and stick representation of the structure of compound **7** in approximately the same orientation was created in the program AVOGADRO. Steroid rings A-D and the positions of carbon 17 and carbon 3 are labeled.

Supplementary references

1. Barca, G. M. J., et al. (2020). "Recent developments in the general atomic and molecular electronic structure system." The Journal of Chemical Physics 152(15): 154102. (doi: 10.1063/5.0005188).

2. Perri, M. J. and S. H. Weber (2014). "Web-Based Job Submission Interface for the GAMESS Computational Chemistry Program." Journal of Chemical Education 91(12): 2206-2208. (doi:10.1021/ed5004228).

3. Hanson, R. M., et al. (2013). "JSmol and the Next-Generation Web-Based Representation of 3D Molecular Structure as Applied to Proteopedia." Israel Journal of Chemistry 53(3-4): 207-216. (doi:10.1002/ijch.201300024).
